# Discovery of a Potent and Highly Isoform-Selective Inhibitor of the Neglected Ribosomal Protein S6 Kinase Beta 2 (S6K2)

**DOI:** 10.3390/cancers13205133

**Published:** 2021-10-13

**Authors:** Stefan Gerstenecker, Lisa Haarer, Martin Schröder, Mark Kudolo, Martin P. Schwalm, Valentin Wydra, Ricardo A. M. Serafim, Apirat Chaikuad, Stefan Knapp, Stefan Laufer, Matthias Gehringer

**Affiliations:** 1 Department of Pharmaceutical/Medicinal Chemistry, Institute of Pharmaceutical Sciences, Eberhard Karls University Tübingen, 72076 Tübingen, Germany; stefan.gerstenecker@uni-tuebingen.de (S.G.); lisa.haarer@uni-tuebingen.de (L.H.); mark.kudolo@uni-tuebingen.de (M.K.); valentin.wydra@uni-tuebingen.de (V.W.); ricardo.serafim@mnf.uni-tuebingen.de (R.A.M.S.); stefan.laufer@uni-tuebingen.de (S.L.); 2 Department of Biochemistry, Chemistry and Pharmacy, Institute for Pharmaceutical Chemistry, Goethe University Frankfurt, 60438 Frankfurt, Germany; m.schroeder@pharmchem.uni-frankfurt.de (M.S.); schwalm@pharmchem.uni-frankfurt.de (M.P.S.); chaikuad@pharmchem.uni-frankfurt.de (A.C.); knapp@pharmchem.uni-frankfurt.de (S.K.); 3 Structural Genomics Consortium, Buchmann Institute for Molecular Life Sciences, Goethe University Frankfurt, 60438 Frankfurt, Germany; 4 German Translational Cancer Network (DKTK) Site Frankfurt/Mainz, Frankfurt Cancer Institute (FCI), 60596 Frankfurt, Germany; 5 Cluster of Excellence iFIT (EXC 2180) ‘Image-Guided & Functionally Instructed Tumor Therapies’, Eberhard Karls University Tübingen, 72076 Tübingen, Germany; 6 Tübingen Center for Academic Drug Discovery, Eberhard Karls University Tübingen, 72076 Tübingen, Germany

**Keywords:** protein kinases, chemical probes, S6K2, p70S6K2, p70S6Kb, RPS6KB2, covalent inhibitors

## Abstract

**Simple Summary:**

The two human p70 ribosomal S6 kinases, S6K1 and S6K2, have been associated with a variety of cellular processes and human pathologies, especially cancer. Thus far, only S6K1 was thoroughly studied and selectively addressed by small molecule inhibitors. Despite growing evidence suggesting S6K2 as a promising anticancer target, this isoform has been severely neglected, which can partly be attributed to the lack of isoform-selective inhibitors to study its function. By exploiting a cysteine residue exclusive to S6K2, we were able to generate the first known isoform-selective S6K2 inhibitor. Besides its excellent selectivity against S6K1 and other human kinases, the compound showed weak intrinsic reactivity and promising in vitro metabolic stability. Our proof-of-concept study provides a basis for the development of high quality S6K2 chemical probes to validate this kinase as a target for therapeutic interventions.

**Abstract:**

The ribosomal protein S6 kinase beta 2 (S6K2) is thought to play an important role in malignant cell proliferation, but is understudied compared to its closely related homolog S6 kinase beta 1 (S6K1). To better understand the biological function of S6K2, chemical probes are needed, but the high similarity between S6K2 and S6K1 makes it challenging to selectively address S6K2 with small molecules. We were able to design the first potent and highly isoform-specific S6K2 inhibitor from a known S6K1-selective inhibitor, which was merged with a covalent inhibitor engaging a cysteine located in the hinge region in the fibroblast growth factor receptor kinase (FGFR) 4 via a nucleophilic aromatic substitution (S_N_Ar) reaction. The title compound shows a high selectivity over kinases with an equivalently positioned cysteine, as well as in a larger kinase panel. A good stability towards glutathione and *N*α-acetyl lysine indicates a non-promiscuous reactivity pattern. Thus, the title compound represents an important step towards a high-quality chemical probe to study S6K2-specific signaling.

## 1. Introduction

Protein kinases have become highly important drug targets during the past 20 years, with over 60 protein kinase inhibitors already being approved by the U.S. Food and Drug Administration (FDA) [1,2]. Despite this success, many of the more than 500 protein kinases of the human kinome remain poorly characterized, and most of the research being carried out focuses on a relatively small set of already well-understood and validated targets [3,4,5]. This narrow scope is not necessarily due to a lack of interest, but is often caused by a lack of enabling tools, such as potent and highly selective inhibitors, for understudied kinases. Pharmacological modulators are required to complement genetic approaches for understanding the roles of neglected kinases and other proteins in signal transduction [6,7]. The growing awareness about this need culminated in the goals set by representatives of academia, industry, and public funders to generate pharmacological modulators for almost all human proteins by 2035 (Target 2035) [7].

The ribosomal protein S6 kinase beta 2 (S6K2 or p70S6Kβ) is an understudied member of the family of ribosomal protein S6 kinases (S6K), which are part of the group of AGC serine/threonine kinases and known to phosphorylate the 40S ribosomal S6 protein. It is encoded by the *RPS6KB2* gene on chromosome 11 and acts as a downstream effector of the AKT/mTOR and RAS/RAF/MEK/ERK pathways [8]. As such, it is involved in the regulation of cell growth and survival. S6K2 usually expresses in low levels, but has been shown to be overexpressed in different forms of cancer, including breast and prostate cancer [9,10]. More specifically, the amplification and gain of S6K2 correlates with breast cancer cells being estrogen-receptor-positive (ER-positive) and has been associated with chemoresistance and a significantly reduced recurrence-free survival [11]. The silencing of the *RPS6KB2* gene provided some insight into its function in regulating malignant cell proliferation and suggested a potential role in cancer therapy. According to these studies, the silencing of S6K2 was able to decrease the cell viability of small-cell lung cancer (SCLC) cells, as well as non-small-cell lung cancer (NSCLC) cells, either via preventing the formation of the FGF2-inducible PKCε/B-RAF/S6K2-complex or the downregulation of the Hedgehog/GLI pathway, respectively [12,13]. Similarly, the knockdown of S6K2 promoted cell death in certain breast cancer cell lines, as well as prostate cancer cell lines [10,14]. However, the effects of the pharmacological inhibition of S6K2 kinase activity remain unknown. Apart from malignant disorders, S6K2 showed an increased phosphorylation suggesting an increased activity during SARS-CoV-2 infection in a cell-based proteomics study [15].

S6K2, which has been denominated as the neglected member of the S6K family [8], shares ~80% of the amino acid sequence in the kinase domain with its closely related and more thoroughly studied homolog S6K1 (also known as p70S6K). Adding to that, other regions involved in regulation, such as the neighboring kinase extension, as well as the pseudo-substrate and inhibitory domain, are also mostly conserved among the two enzymes [16,17]. Differences can be found mainly in their C- and N-terminal region. The only significant difference in the ATP binding site, however, is a non-catalytic cysteine (Cys_150_; see the alignment in Figure S1) in the hinge region of S6K2 (Tyr_174_ in S6K1), which is only found in four other human protein kinases, namely MAPKAPK2, MAPKAPK3, FGFR4, and TTK [18].

It is believed that S6K1 and S6K2 share some biological functions, but also have isoform-specific roles. S6K2 seems to be more involved in regulating cell death compared to S6K1, which has a more marked role in cell proliferation, invasion, and metastasis. In one example, the knockdown of S6K2 led to cell death in breast cancer cells, whereas the knockdown of S6K1 led to the activation of other pathways and ultimately to the inhibition of apoptosis, which corroborates the distinct roles of the underlying signaling networks [9]. The overall scarcity of S6K2-focused research prompted several authors to call for more extensive efforts in this field, especially focusing on pharmacological modulators and molecular probes [9,19,20].

So far, only two S6K1 inhibitors with notable selectivity over S6K2 in biochemical assays have been reported (FL772 [21] and PF-4708671 [22]), and no S6K2-selective inhibitor is known [23]. The minor structural differences in the kinase domain of S6K2 and S6K1 make it difficult to target either one of these isoforms by small molecules selectively. In our work, we aimed to exploit the presence of Cys_150_ in the hinge region of S6K2 to achieve a high inhibitory potency and excellent (isoform-)selectivity by means of a covalent reactive group (“warhead”). For this purpose, we wanted to make use of an electron-deficient heteroaryl system equipped with a leaving group to react with Cys_150_ via nucleophilic aromatic substitution (S_N_Ar). The S_N_Ar strategy extends the scope of cysteine-targeted warheads beyond the typical α,β-unsaturated carbonyl compounds, and may be able to overcome some of their limitations in terms of tunability, sterics, and metabolic properties [24,25,26]. However, such an approach has, to the best of our knowledge, only been reported once in the context of kinase inhibitor discovery, with screening hit **1** (Figure 1a) from Fairhurst et al. targeting a cysteine in the middle-hinge region of fibroblast growth factor receptor kinase (FGFR) 4 via a unique binding mode [27]. The equivalent positioning of the FGFR4 cysteine compared to S6K2 Cys_150_, along with the presence of a larger tyrosine residue at the same position in S6K2, suggested that an analogous strategy may also be accomplished to generate isoform-selective S6K2 inhibitors.

## 2. Materials and Methods

### 2.1. Molecular Modeling

Pairwise sequence alignments were generated with the EMBOSS Needle online tool (https://www.ebi.ac.uk/Tools/psa/emboss_needle/, accessed on 29 September 2021) using the sequences of S6K1 (form alpha I) and S6K2 (form I). Molecular modeling was performed using the Schrödinger Small-Molecule Drug Discovery Suite 2017-3 (Schrödinger, LLC, New York, NY, USA). For covalent docking, the CovDock module was employed in the pose prediction mode using standard settings. The figures were generated with PyMOL 1.8.2.0. (Schrödinger, LLC, New York, NY, USA).

### 2.2. Chemistry

All starting materials and reagents were of commercial quality and were used without further purification. Thin layer chromatography (TLC) was carried out on Merck 60 F254 silica plates (Merck KGaA, Darmstadt, Germany) and visualized under UV light (254 nm and 366 nm) or developed with an appropriate staining reagent. Preparative column chromatography was carried out with an Interchim PuriFlash 430 or PuriFlash XS420 (Interchim S.A., Montlucon, Allier, France) automated flash chromatography system using normal phase Grace Davison Davisil LC60A 20–45 micron silica (W.R. Grace and Company, Columbia, MD, USA) or Merck Geduran Si60 63–200 micron silica (Merck KGaA, Darmstadt, Germany). For synthetic procedures and synthesis schemes (Schemes S1 and S2), see the Supplementary Information.

Nuclear magnetic resonance (NMR) spectral analysis was performed on Bruker Avance III HD 600/400/200 instruments (Bruker Corporation, Billerica, MA, USA). The samples were dissolved in deuterated solvents and chemical shifts were given in relation to tetramethylsilane (TMS). The multiplicity of signals was indicated with s = singlet, d = doublet, dd = doublet of doublets, t = triplet, q = quartet, br = broad singlet, and m = multiplet. Spectra were calibrated using the residual peaks of the used solvent. For the NMR-spectra of the title compound **2** and the unreactive control compound **21** please see the Supporting Information Figures S3–S5 and Figures S6–S8, respectively. Mass spectrometry (MS) was carried out with an Advion TLC-MS interface (Advion, Ithaca, NY, USA) with electrospray ionization (ESI) in positive and/or negative mode. Instrument settings were as follows: ESI voltage 3.50 kV, capillary voltage 187 V, source voltage 44 V, capillary temperature 250 °C, desolvation gas temperature 250 °C, and gas flow 5 L/min nitrogen. HPLC purity was determined on an Agilent 1100 series (degasser, binary pump, injection module and ColCom setup, Agilent Technologies Inc., Santa Clara, CA, USA) with a 1260 DAD detector module. HPLC method for purity assessment was as follows: Phenomenex Kinetex^®^ 2.6 µm C8 100 Å 150 × 4.6 mm column (Phenomenex Inc., Torrance, CA, USA), injection volume: 5 μL, flow rate: 0.5 mL/min at 23 °C; 0 min: 40% MeOH, 60% phosphate buffer pH 2.3; 15 min: 85% MeOH, 15% phosphate buffer pH 2.3; 20 min: 85% MeOH, 15% phosphate buffer pH 2.3; 22 min: 40% MeOH, 60% phosphate buffer pH 2.3; 28 min: 40% MeOH, 60% phosphate buffer pH 2.3.

### 2.3. Glutathione (GSH) Stability Assay

The performed GSH stability assay for the title compound **2** and Afatinib was based on the GSH assay established by Keeley et al. [28] for heterocyclic electrophilic fragments. The following deviations from the original protocol were made: the reaction medium was changed to MeCN/PBS-buffer 50:50 due to limited solubility of **2** in aqueous buffer. The reaction temperature was chosen to be 40 °C. The reaction of **2** with GSH was monitored by measuring the decreasing area under the curve (AUC) of the compound relative to the internal standard indoprofen. The compound **2** + GSH adduct was later synthesized under the assay conditions at a larger scale and characterized by HRMS (ESI(+) calcd. for [M + H]^+^: *m*/*z* = 833.27649; found: 833.27616) to allow for assignment in the HPLC chromatograms via retention time and DAD spectra.

### 2.4. Nα-Acetyl Lysine Stability Assay

The *N*α-acetyl lysine stability assay was performed in analogy to the GSH stability assay by substituting GSH with *N*α-acetyl lysine at pH 7.4 and additionally at pH 10.2 to mimic the p*K*_a_ depression often observed for biologically relevant lysines [29]. PBS-buffer was substituted with a sodium tetraborate buffer (28 mM) for experiments at pH 10.2. The compound **2** + *N*α-acetyl lysine adduct was characterized by LC-HRMS (ESI(+) calcd. for [M + H]^+^: *m*/*z* = 714.3081; found: 714.3019).

### 2.5. Biochemical Assays

The provided IC_50_ values were measured using the HotSpot^TM^ Kinase Profiling assay and conducted at Reaction Biology Corp. in their facilities in Malvern, PA, USA. All IC_50_ values were first determined by singlicate measurements starting at a concentration of 5 μM with a 10-fold serial dilution (5-point measurement) and triplicates were subsequently determined for S6K2 starting at 0.1 μM with 4-fold serial dilution to obtain a more precise value.

### 2.6. Microsomal Stability

Pooled liver microsomes from mice (male) were purchased from XenoTech LLC (Sekisui XenoTech LLC, Kansas City, KS, USA). Incubation of compound **2** was made in the presence of an NADPH-regenerating system (5 mM glucose-6-phosphate, 5 U/mL glucose-6-phosphate dehydrogenase, and 1 mM NADP^+^). Compound **2** (100 μM), the NADPH-regenerating system, and 4 mM MgCl_2_⋅6 H_2_O in 0.1 M Tris buffer (pH 7.4) were preincubated for 5 min at 37 °C and 750 rpm on a shaker. The incubation mix was split into aliquots (50 μL) and the reaction was started by the addition of mouse liver microsomes. The reaction was quenched at selected time points (0, 10, 20, 30, 60, and 90 min) by adding 100 μL internal standard at a concentration of 50 μM in MeCN. The samples were vortexed for 30 s and centrifuged (19,800 relative centrifugal force/4 °C/15 min). The supernatant was directly used for LC-MS analysis. All incubations were conducted in triplicates and a limit of 1% organic solvent was not exceeded.

Sample separation was performed on an Alliance 2695 HPLC (Waters GmbH, Eschborn, Germany) equipped with a Phenomenex Kinetex, 2.6 µm, C18, 100 Å, 100 × 3.00 mm column with a 15 min gradient (Phenomenex Inc., Torrance, CA, USA). Mobile phase A: 90% water, 10% acetonitrile, and additional 0.1% formic acid (*v*/*v*); mobile phase B: acetonitrile with 0.1% formic acid (*v*/*v*). The gradient was set to: 0–2.5 min 10% B, 2.5–12.5 min from 10% to 50% B, 12.5–15 min 50% B, 15.01–18 min from 50% to 10% B at a flow rate of 0.6 mL/min. Samples were maintained at 10 °C and the column temperature was set to 40 °C with an injection volume of 5 μL.

Detection was performed on a Micromass Quattro micro triple quadrupole mass spectrometer (Waters GmbH, Eschborn, Germany) using electrospray ionization in positive mode. Spray, cone, extractor, and RF lens voltages were set to 4 kV, 30 V, 5 V, and 1 V, respectively. The desolvation temperature was set to 350 °C and the desolvation gas flow was at 650 L/h. The data were analyzed using MassLynx 4.1 (Waters Corporation, Milford, MA, USA)

### 2.7. Differential Scanning Fluorimetry (DSF) Assay

Differences in the melting temperature (ΔT_m_) were measured as described by Fedorov et al. [30]. Purified proteins were buffered in 25 mM HEPES (pH 7.5), 500 mM NaCl and were assayed in a 384-well plate with a final protein concentration of 2 μM in 10 μL assay volume. Inhibitors were added to a final concentration of 10 μM using an ECHO 550 acoustic dispenser (Labcyte, San José, CA, USA). As a fluorescence probe, SYPRO-Orange (Molecular Probes, Eugene, OR, USA) was added in a 1:5000 dilution. Filters for excitation and emission were set to 465 nm and 590 nm, respectively. The temperature was increased from 25 °C with 3 °C/min to a final temperature of 95 °C, while scanning, using the QuantStudio5 (Applied Biosystems, Waltham, MA, USA). Data were analyzed through Boltzmann equation in the Protein Thermal Shift software (Applied Biosystems, Waltham, MA, USA). Samples were measured in technical duplicates.

## 3. Results and Discussion

### 3.1. Design

**Figure 1:**
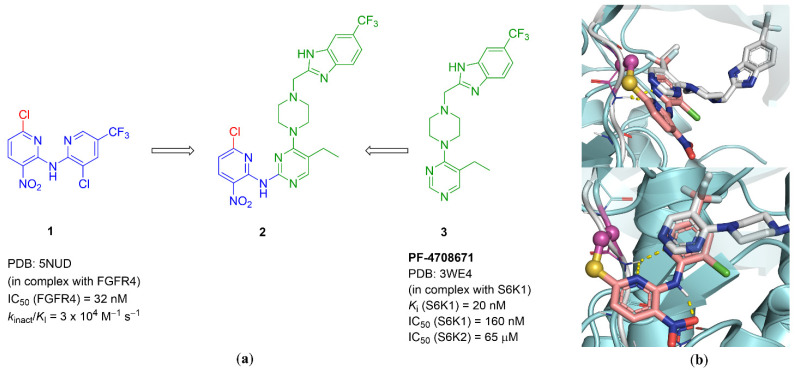
(**a**) Merging of PF-4708671 (**3**, green) [31] with compound **1** (blue) [27] containing a reactive chloronitropyridine (leaving group in red); (**b**) crystal structure overlay of FGFR4 in complex with compound **1** (salmon; FGFR4 backbone in cyan) and S6K1 in complex with PF-4708671 (grey). The dotted yellow lines indicate the supposed chelate-type hydrogen bond formed by compound **1**, as well as the intramolecular hydrogen bond between the nitro group and the diaryl-*NH*. The FGFR4 cysteine (Cys_552_) is highlighted in magenta.

Our design strategy started from the co-crystal structure of the known reversible S6K1 inhibitor PF-4708671 (**3**, Figure 1a, IC_50_ = 160 nM), which was reported to possess a weak off-target activity on S6K2 (IC_50_ = 65 μM) [22]. We aimed to attach the reactive chloronitropyridine fragment of FGFR4 inhibitor **1** (Figure 1a) to the hinge-binding core of compound **3** via an amino linker in order to mimic the binding mode of the template inhibitor. Since no X-ray crystal structure of S6K2 is available so far, we created a hinge-region-centered structure overlay of compound **1** bound to FGFR4 (PDB: 5NUD [27]) and PF-4708671 (**3**) bound to S6K1 (PDB: 3WE4 [31]) (Figure 1b). The superimposition indeed showed a good structural alignment of the core scaffolds and indicated that the thiol group of Cys_150_ in S6K2 should be reachable by the chloronitropyridine warhead. This hypothesis was corroborated by covalent docking into a S6K1 Y174C mutant generated in silico (see Figure S2). Interestingly, the distance between the *NH* of Ala_553_ in FGFR4 and the two pyridine nitrogen atoms of compound **1** (Figure 1b) suggests the formation of a chelate-like hydrogen bond. The latter, together with a weak intramolecular hydrogen bond involving the nitro group [32] to mask the diaryl-*NH* from interacting with the hinge region, may be important to both favor a suitable pre-orientation and to further activate the electron-deficient aryl system facilitating the supposed displacement reaction.

### 3.2. Synthesis

**Scheme 1:**
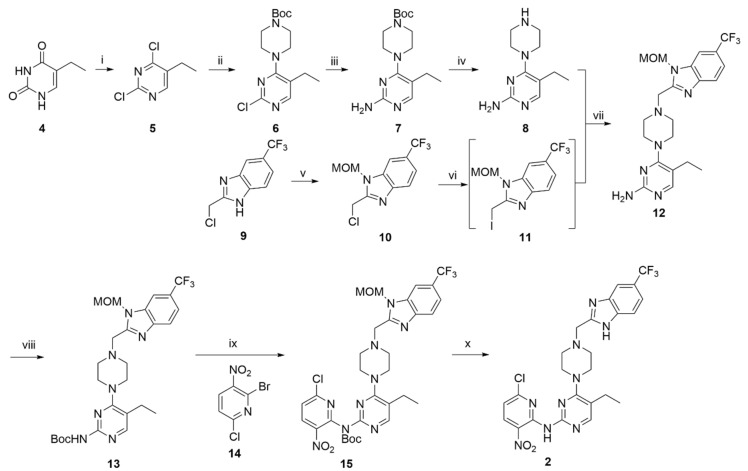
Synthesis of the title compound **2**. Reagents and conditions: (**i**) POCl_3_, DIPEA, reflux, 4 h, 98%; (**ii**) Boc-piperazine, EtOH, −20 °C, 17 h, 82%; (**iii**) LiHMDS, RuPhos Pd G4 2.5 mol%, 1,4-dioxane, 50 °C, 1 h, quant.; (**iv**): HCl, 1,4-dioxane, reflux, 1 h, quant.; (**v**) MOMBr, DIPEA, THF, −40 °C, 17 h, 68%, 10:8.7 regioisomer ratio; (**vi**) NaI, acetone, rt, 30 min in situ; (**vii**) DIPEA, acetone, 0 °C to rt, 17 h, 80% (**viii**) Boc_2_O, *t*BuOH, 40 °C, 17 h, quant.; (**ix**) **14**, Cs_2_CO_3_, XantPhos Pd G4 5 mol%, toluene, 55 °C, 3 d, 69%; (**x**) HCl, 1,4-dioxane, reflux, 2 h, 88%. Quant., quantitative yield; DIPEA, *N*,*N*-diisopropylethylamine; Boc, *tert*-butyloxycarbonyl; LiHMDS, lithium bis(trimethylsilyl)amide; RuPhos Pd G4, 2-dicyclohexylphosphino-2′,6′-diisopropoxybiphenyl 4th generation Buchwald palladacycle catalyst; THF, tetrahydrofuran; MOMBr, bromomethyl methyl ether; rt, room temperature; Boc_2_O, di-*tert*-butyl dicarbonate; XantPhos Pd G4, 4,5-bis(diphenylphosphino)-9,9-dimethylxanthene 4th generation Buchwald palladacycle catalyst.

The synthesis of the title compound **2** is summarized in Scheme 1, and started from the commercially available uracil derivative **4,** which could be chlorinated by POCl_3_ in the presence of DIPEA [33]. The attachment of the Boc-protected piperazine onto product **5** was achieved by a S_N_Ar reaction under temperature-controlled conditions, with the almost exclusive formation of the desired regioisomer **6**. The introduction of the amino group to deliver the 2-aminopyrimidine **7** was possible via a Buchwald–Hartwig arylamination with LiHMDS using the RuPhos Pd G4 precatalyst, after several common ammonia surrogates failed to provide useful amounts of the product under catalyzed and non-catalyzed conditions. The deprotection of intermediate **7** by means of acid led to free piperazine **8**. Chloromethylbenzimidazole **9** was equipped with a MOM-protecting group (**10**) prior to nucleophilic displacement by piperazine **8** to prevent side reactions observed for unprotected benzimidazole **9** in the presence of a base. The nucleophilic substitution was best carried out under Finkelstein conditions via iodomethylbenzimidazole **11** being generated in situ, with much lower yields being observed for the direct substitution with the respective chloro-derivative **10**. Originally, we planned to directly connect the core scaffold **12** with the bromo-substituted proto-warhead **14** under Buchwald–Hartwig conditions. After screening a variety of different catalysts, we found XantPhos to be the only tested phosphine ligand suitable to enable the *C*-*N* bond formation. Surprisingly, we observed that the reaction mainly furnished the double-*N*-arylated product, even when only one equivalent of proto-warhead **14** was used. This behavior is thought to arise from the increased *NH*-acidity of the coupling product, which may overcompensate the decrease in reactivity caused by the steric hindrance in the mono-*N*-arylated intermediate. We therefore hypothesized that the aminopyrimidine **13** carrying an electro-withdrawing Boc-protecting group may also react under these conditions. To our delight, we found that the Buchwald–Hartwig coupling of intermediate **13** with the proto-warhead **14** delivered monoarylated product **15** in a good yield, which could then be globally deprotected to obtain final compound **2**.

**Scheme 2:**
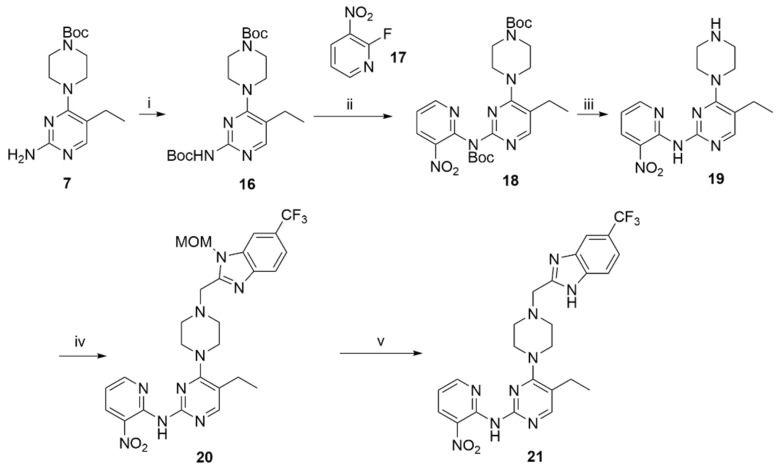
Synthesis of the unreactive analog **21**. Reagents and conditions: (**i**) Boc_2_O, *t*BuOH, 40 °C, 2 h, 80%; (**ii**) NaH, DMF, 0 °C to rt, 1 h, then 2-fluoro-3-nitropyridine (**17**), DMF, rt, 7 d, 62%; (**iii**) HCl, 1,4-dioxane, 50 °C, 4 h, 89%; (**iv**) **10**, NaI, acetone, rt, 30 min then **19**, DIPEA, acetone, 0 °C to rt, 17 h, 68%; (**v**) HCl, 1,4-dioxane, 50 °C, 4 h, 80%. Boc_2_O, di-*tert*-butyl dicarbonate; DMF, dimethylformamide; rt, room temperature, DIPEA, *N,N*-diisopropylethylamine.

The synthesis of the unreactive analog **21** lacking the chloride leaving group started from the common precursor **7**, but was modified so that the benzimidazole moiety could be attached at a later stage, allowing for easier derivatization in future endeavors (Scheme 2). Aminopyrimidine **7** was Boc-protected to intermediate **16** and subjected to a S_N_Ar-reaction with 2-fluoro-3-nitropyridine (**17**) after deprotonation with sodium hydride to afford diarylamine **18**. Both Boc-protecting groups were cleaved under acidic conditions to give the free piperazine **19**. Then, the benzimidazole-derived side chain was attached via nucleophilic displacement under Finkelstein conditions, as described before. Such a late-stage introduction of the side chain was not possible for the title compound **2**, since the piperazine would attach to the reactive warhead. The cleavage of the MOM-protecting group in **20** yielded the non-reactive control compound **21**.

### 3.3. Biochemical Evaluation

In accordance with our design strategy, title compound **2** proved to be highly potent in an enzymatic assay, with an apparent IC_50_ of 22 nM for S6K2 (see Figure S12), while being inactive for the closely related isoform S6K1 at a maximum tested compound concentration of 5 μM (Table 1). To investigate if the formation of a covalent bond between Cys_150_ and inhibitor **2** could drive the observed S6K2 potency, we tested unreactive analog **21** lacking the leaving group. This compound showed negligible inhibitory activity for S6K2 (IC_50_ > 5000 nM). These findings, in combination with the fact that no other nucleophilic moieties differ between the S6K1 and S6K2 ATP binding site (see Figure S1), support the notion of inhibitor **2** being able to bind covalently to S6K2. A key factor contributing to the high isoform-selectivity of compound **2** might also be the increased steric hindrance of Tyr_174_ in S6K1 compared to Cys_150_ and the resulting repulsion of the substituted pyridyl group. Notably, this difference in sterics may also be exploited for the rational design of isoform-selective reversible S6K2 inhibitors.

The IC_50_ values of compound **2** for MAPKAPK2, MAPKAPK3, FGFR4, and TTK were also determined (see Table 1). The compound did not show significant inhibitory activity on any of those kinases, except for FGFR4 (IC_50_ = 216 nM, see Figure S12), confirming a good selectivity against kinases with an equivalent cysteine. To explore the broader kinome selectivity, a thermal shift screen (see Table S1 and Figure S14) was performed. Of the 97 kinases and three bromodomains included in this panel, only MAP2K4 (MKK4) showed a significant ΔT_m_ > 2 °C (S6K2 was not tested). Given the even distribution of the kinases in the panel across all major kinase families, a favorable selectivity of compound **2** can be assumed.

To test whether the biological activity of the title compound **2** stemmed from a potential non-specific reactivity, we evaluated it in a HPLC-based glutathione (GSH) reactivity assay [28] in comparison to the FDA-approved covalent kinase inhibitor Afatinib featuring a common acrylamide-derived warhead (see Figures S9 and S10). Inhibitor **2** showed a >10-fold longer half-life than Afatinib at physiological pH and a 20-fold excess of GSH (5 mM), suggesting a favorable reactivity range for specific covalent targeting. Inhibitor **2** was further tested in an assay assessing its reactivity towards *N*α-acetyl lysine, where it showed negligible reactivity at physiological pH and a slightly increased reactivity at pH 10.2 (*t*_1/2_ = 27 h; see Figure S11). The low intrinsic reactivity in both assays is in agreement with the clean profile observed in the kinase panel. Notably, a major benefit of the S_N_Ar-based warhead lies in the highly adjustable reactivity of such electrophiles, which can be modulated by varying the substitution pattern of the aryl system, ring size, number of annellated systems, and heteroatom content [35], allowing for further fine-tuning if required. Compound **2** also showed an acceptable stability in mouse liver microsomes, with no signs of the nitro group being a metabolic hotspot (see Figure S13). The observed main metabolites had molecular weights differing by −2 Da and +16 Da, which might be attributed to iminium formation via oxidation at the piperazine ring and subsequent ring-opening hydrolysis.

## 4. Conclusions

In summary, we have designed the first isoform-selective inhibitor of S6K2 (**2**) by merging the structures of S6K1 inhibitor **3** and covalent FGFR4 inhibitor **1**. The incorporation of an electrophilic aromatic system known to bind covalently to a cysteine in the hinge region of FGFR4, which is also present in S6K2, was the key to success. A synthesis route was developed to efficiently deliver key compound **2**, which will also allow for future structure–activity relationship (SAR) exploration. Our inhibitor **2** showed a high biochemical potency and selectivity for S6K2. The selectivity over the closely related isoform S6K1 can be rationalized by the supposed formation of a covalent bond with Cys_150_ of S6K2, which is a tyrosine in S6K1. Although we cannot fully rule out that the observed S6K2 potency relies on a strong contribution of the chloro substituent to the reversible binding affinity, the hypothesized covalent binding mode is in accordance with the poor S6K2 inhibitory activity of the unreactive control compound **21**, suggesting that the potency of compound **2** may mainly be driven by efficient covalent inactivation rather than by strong reversible binding. Nevertheless, the intrinsic reactivity of inhibitor **2** was low and we found no indications of the chloronitropyridine warhead being a hotspot of hepatic metabolism. Future studies will focus on the characterization of the suggested covalent mode-of-action, improvement of the (reversible) binding affinity, and further exploration of warhead chemistry. The binding affinity may be increased, for example, by employing alternative hinge-binding motifs capable of anchoring the compound via an additional hydrogen bond, by further filling the pocket occupied by the pyrimidine ethyl substituent or by rigidization approaches, whereas the replacement of the nitro group by more drug-like moieties would be a main goal on the warhead side. With the clean thermal shift kinome profile and FGFR4 being the only off-target among the kinases with an equivalent cysteine (MAPKAPK2, MAPKAPK3, FGFR4, and TTK), we expect inhibitor **2** to serve as an excellent starting point for the generation of highly potent and specific chemical probes for studying S6K2 function in vitro and in vivo.

## Figures and Tables

**Table 1:** Results of the biochemical kinase assays.

No.	S6K2 IC_50_ [nM]	S6K1 IC_50_ [nM]	MAPKAPK2 IC_50_ [nM]	MAPKAPK3 IC_50_ [nM]	FGFR4 IC_50_ [nM]	TTK IC_50_ [nM]
**2**	22 ± 1.6	>5000	>5000	>5000	216	>5000
**21**	>5000	ND	ND	ND	ND	ND

Shown IC_50_ values were commercially determined by five-point singlicate measurements, with the exception of compound **2** for S6K2 (five-point triplicate; see the Supporting Information), at ReactionBiology Corp. using the Hotspot^TM^ platform [34]. ± Standard deviation. ND: not determined.

## Data Availability

Data are contained within the article or supplementary material.

## Supplementary Materials

The following are available online at https://www.mdpi.com/article/10.3390/cancers13205133/s1, Scheme S1: Synthesis of the title compound **2**, Scheme S2: Synthesis of the unreactive analog **21**, Figure S1: Sequence alignment of S6K1 and S6K2 and structural overlay of the kinase domains of S6K1 and S6K2, Figure S2: Covalent docking of compound **2** into an S6K1-Y174C in silico mutant, Figure S3: ^1^H-NMR spectrum of **2** in CDCl_3_, Figure S4: ^1^H-NMR spectrum of **2** in DMSO-d_6_, Figure S5: ^13^C-NMR spectrum of **2** in DMSO-d_6_, Figure S6: ^1^H-NMR spectrum of **21** in CDCl_3_, Figure S7: ^1^H-NMR spectrum of **21** in DMSO-d_6_, Figure S8: ^13^C-NMR spectrum of **21** in DMSO-d_6_, Figure S9: GSH stability assay of compound **2**, Figure S10: GSH stability assay of Afatinib, Figure S11: *N*α-acetyl lysine stability assay of compound **2** at pH 7.4 and pH 10.2, Figure S12: IC_50_ curves for the title compound **2**, Figure S13: Microsomal stability of compound **2**, Figure S14: Protein kinases assessed in the DSF screening panel, Table S1: Protein kinase thermal shift assay with ΔT_m_ values for title compound **2**.
