# Supplementary material for: Discovery of a Potent and Highly Isoform-Selective Inhibitor of the Neglected Ribosomal Protein S6 Kinase Beta 2 (S6K2)
Source: Cancers (Basel). 2021 Oct 13;13(20):5133. doi: 10.3390/cancers13205133 (PMC8534050; doi:10.3390/cancers13205133)
Supplement: Supplementary file 1 [file cancers-13-05133-s001.zip › cancers-1381363-supplementary.pdf]

Supplementary Materials

Discovery of a Potent and Highly Isoform-Selective Inhibitor of the Neglected Ribosomal Protein S6 Kinase Beta 2 (S6K2)

Stefan Gerstenecker, Lisa Haarer, Martin Schröder, Mark Kudolo, Martin P. Schwalm, Valentin Wydra, Ricardo A.M. Serafim, Apirat Chaikuad, Stefan Knapp, Stefan Laufer and Matthias Gehringer

1. Modeling and Sequence Alignments

1.1. Sequence and Structural Alignment of S6K1 and S6K2

```
Sequence alignment of S6K1 (Isoform alpha 1) and S6K2 (Isoform 1) generated with
EMBOSS Needle Pairwise Sequence Alignment

COLOR CODE:

LYSINES
CYSTEINES
S6K2 specific LYSINES
S6K2 specific CYSTEINES
TARGET CYSTEIN

Residues with side chain contributions to the ATP binding pocket are highlighted in
bold letters. The kinase domain is underlined.

S6K1                 1 MRRRRRRDGFYPAPDFRDREAEDMAGVFDIDLDQPEDAGSEDELE----E     46
                                              ||.|||:||:..|.:..|.|.|    :
S6K2                 1 -----------------------MAAVFDLDLETEEGSEGEGEPELSPAD     27

S6K1                47 GGQLNESMDHGGVGPYELGMEHCEKFEISETSVNRGPEKIRPECFELLRV     96
                       ...|.| :...|:.|    :.|.|:.|::|||||.|||:|.|.|||||||
S6K2                28 ACPLAE-LRAAGLEP----VGHYEEVELTETSVNVGPERIGPHCFELLRV     72

S6K1                97 LGKGGYGKVFQVRKVTGANTGKIFAMKVLKKAMIVRNAKDTAHTKAERNI    146
                       ||||||||||||||.|.|.|||:|||||:||.||||||||||||:|||||
S6K2                73 LGKGGYGKVFQVRKVQGTNLGKIYAMKVLRKAKIVRNAKDTAHTRAERNI    122

S6K1               147 LEEVKHPFIVDLIYAFQTGGKLYLILEYLSGGELFMQLEREGIFMEDTAC    196
                       ||.||||||:|.||||||||||||||||.|||||||..|||||||:|||||
S6K2               123 LESVKHPFIVELAYAFQTGGKLYLILECLSGGELFTHLEREGIFLEDTAC    172

S6K1               197 FYLAEISMALGHLHQKGIIYRDLKPENIMLNHQGHVKLTDFGLCKESIHD    246
                       ||||||::||||||.:||||||||||||||:.|||:||||||||||||:
S6K2               173 FYLAEITLALGHLHSQGIIYRDLKPENIMLSSQGHIKLTDFGLCKESIHE    222

S6K1               247 GTVTHTFCGTIEYMAPEILMRSGHNRAVDWWSLGALMYDMLTGAPPFTGE    296
                       |.||||||||||||||||:|||||||||||||||||||||||||:||||.|
S6K2               223 GAVTHTFCGTIEYMAPEILVRSGHNRAVDWWSLGALMYDMLTGSPPFTAE    272

S6K1               297 NRKKTIDKILKCKLNLPPYLTQEARDLLKKLLKRNAASRLGAGPGDAGEV    346
                       ||||:|||::.||.||||||.:||||:||.||||.:.|:|.|||||.:|
S6K2               273 NRKKTMDKIIRGKLALPPYLTPDARDLVKKFLKRNPSQRIGGGPGDAADV    322

S6K1               347 QAHPFFRHINWEELLARKVEPPFKPLLQSEEDVSQFDSKFTRQTPVDSPD    396
                       |.||||||:||::|||.:|:|||:|.||||||||||||::|||||||||||
S6K2               323 QRHPFFRHMNWDDLLAWRVDPPFRPCLQSEEDVSQFDTRFTRQTPVDSPD    372

S6K1               397 DSTLSESANQVFLGFTYVAPSVLESVKEKFSFEPKIRSPRRFIGSPRTPV    446
                       |:.||||||||.||||||||||||:|:||.|||:||:|||||...|||.||
S6K2               373 DTALSESANQAFLGFTYVAPSVLDSIKEGFSFQPKLRSPRRLNSSPRAPV    422

S6K1               447 SPVKFSPGDFWGRGASAS----TANPQTPVEYPMETSGIEQMDVTMSGEA    492
                       ||:||||  |.|...|.|    |..|..|:..|...|            .
S6K2               423 SPLKFSP--FEGFRPSPSLPEPTELPLPPLLPPPPPS------------T    458

S6K1               493 SAPLPIRQPNSGPYKKQAFPMISKRPEHLRMNL    525
                       :||||||.|:.....|:.     ..||..
S6K2               459 TAPLPIRPPSGTKKSKRG----RGRPGR-----    482
```

(a)

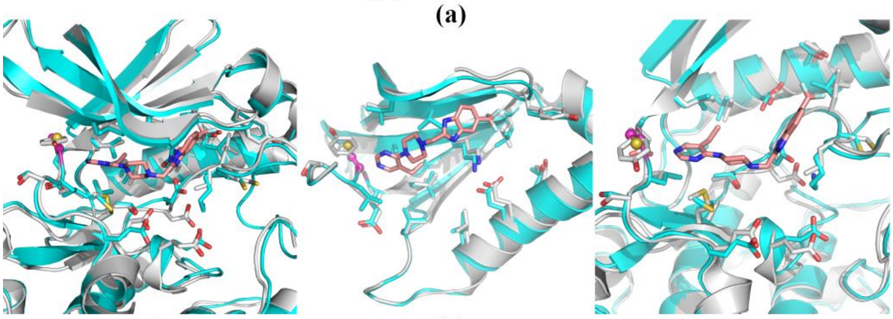

(b) (c) (d)

Figure S1. (**a**): Sequence alignment of S6K1 (form alpha I) and S6K2 (form I) highlighting differences in nucleophilic amino acids (cysteine and lysine), as well as differences in the ATP binding site. (**b**–**d**): Structural overlay of the kinase domains of S6K1 and S6K2. The overlay was generated from an X-ray crystal structure of S6K1 (PDB: 3WE4 [1], grey) and a homology model of S6K2 (cyan) which was generated by SWISS-MODEL (https://swissmodel.expasy.org/repository/uniprot/Q9UBS0, accessed on 3 October 2021) [2] using the S6K1 X-ray crystal structure with the PDB code 4L44 [3] as the template. Amino acids with side chain contributions to the ATP pocket are highlighted as sticks. The only divergent amino acid in this area is Cys_{150} (magenta) in S6K2, being a tyrosine (Tyr_{174}) in S6K1. (**b**): Front view. (**c**): Front/bottom view without the C-lobe. (**d**): Front/top view without sheets β1-β3 from the N-lobe.

1.2. Molecular Modeling

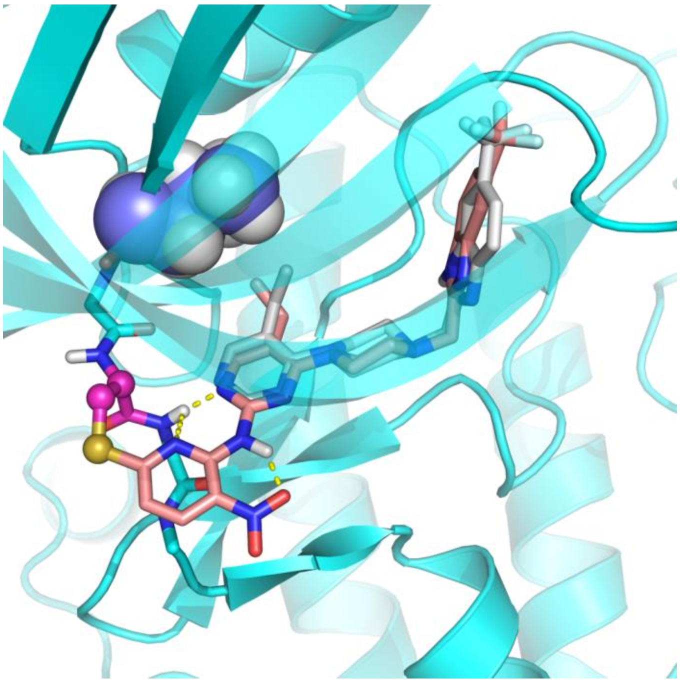

Figure S2. Covalent docking of compound **2** into an S6K1-Y174C in silico mutant. The covalent docking pose of compound **2** (salmon) in S6K1-Y174C was obtained using the CovDock module of the Schrödinger Small Molecule Drug Discovery Suite (2017-3). The in silico mutant was generated from the S6K1 crystal structure with the PDB-code 3WE4, and the highest scoring pose is shown. The docking pose is overlayed with the binding mode of the original ligand (PF-4708671; **3**) from the same template crystal structure. The target cysteine is highlighted in magenta, whereas the leucine gatekeeper residue is shown in purple-blue as space-filling spheres.

2. Synthesis

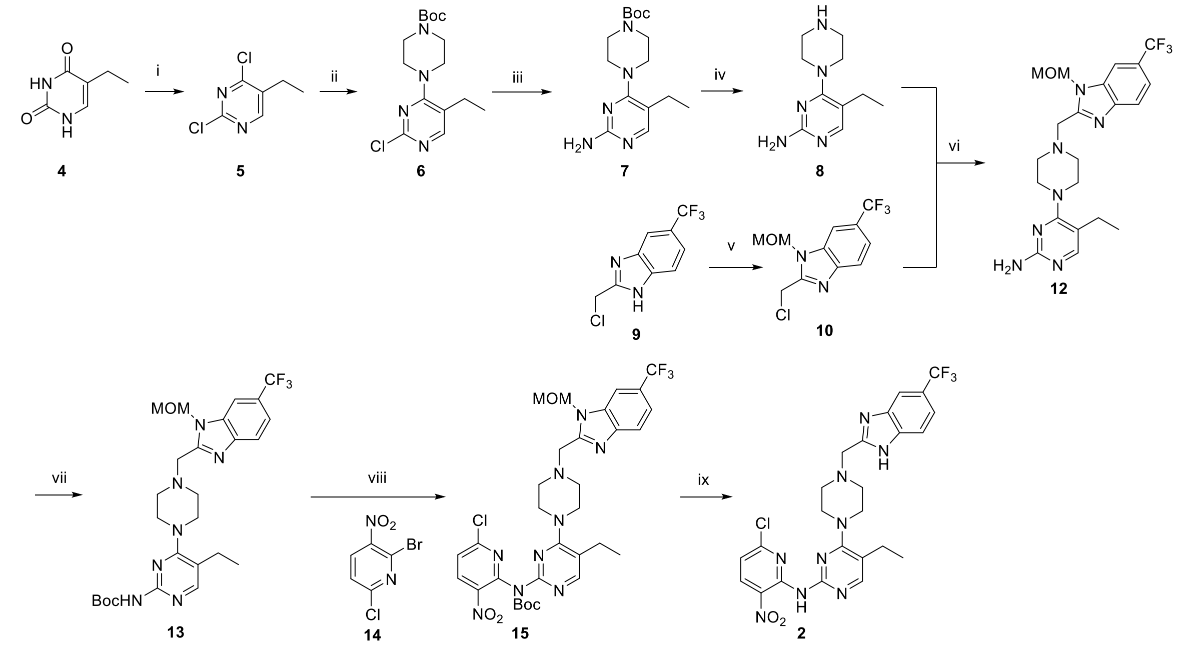

Scheme S1. Synthesis of the title compound **2.**

*2.1. Step i: 2,4-Dichloro-5-ethylpyrimidine (**5**)*

5-Ethylpyrimidine-2,4(1*H*,3*H*)-dione (**4**, 5.00 g, 35.7 mmol) was treated with $POCl_3$ and DIPEA according to the procedure given in WO2004041822A1 [4] to yield 2,4-dichloro-5-ethylpyrimidine (**5**, 6.20 g, 98% yield). ^{1}H-NMR (200 MHz, $CDCl_3$): δ [ppm]: 8.40 (s, 1H), 2.72 (q, J = 7.6 Hz, 2H), 1.26 (t, J = 7.6 Hz, 3H). ^{13}C-NMR (50 MHz, $CDCl_3$): δ [ppm]: 161.9, 159.2, 157.9, 134.0, 23.0, 12.8.

*2.2. Step ii: tert-Butyl 4-(2-chloro-5-ethylpyrimidin-4-yl)piperazine-1-carboxylate (**6**)*

Tert-butyl piperazine-1-carboxylate (6.82 g, 36.6 mmol, 1.10 equiv.) in abs. EtOH (50 mL) at −20 °C was slowly added to a solution of 2,4-dichloro-5-ethylpyrimidine (**5**, 5.90 g, 33.3 mmol, 1.00 equiv.) and DIPEA (5.82 mL, 33.3 mmol, 1.00 equiv.) in abs. EtOH (100 mL). The reaction was stirred at this temperature for 17 h. The mixture was diluted with EtOAc (400 mL) and washed with sat. $NaHCO_3$ (2 x 50 mL). The organic layer was dried over Na_2SO_4 and concentrated. The residue was purified by column chromatography (120 g silica gel, gradient 70–100% EtOAc/hexane, 10 CV) to afford the product *tert*-butyl 4-(2-chloro-5-ethylpyrimidin-4-yl)piperazine-1-carboxylate (**6**, 8.89 g, 82% yield). ^{1}H-NMR (200 MHz, $CDCl_3$): δ [ppm] = 8.09 (s, 1H), 3.76 (dd, J = 6.3, 4.2 Hz, 4H), 3.48 (dd, J = 6.3, 4.2 Hz, 4H), 2.56 (q, J = 7.6 Hz, 2H), 1.48 (s, 9H), 1.18 (t, J = 7.5 Hz, 3H). ^{13}C-NMR (50 MHz, $CDCl_3$): δ [ppm] = 165.3, 158.2, 157.5, 154.7, 121.2, 80.0, 47.6, 43.0, 28.6, 23.3, 13.5. ESI(+) calcd. for $[M+Na]^+$**6**: *m/z* = 349.1; found: 349.0.

*2.3. Step iii: tert-Butyl 4-(2-amino-5-ethylpyrimidin-4-yl)piperazine-1-carboxylate (**7**)*

RuPhos Pd G4 (0.260 g, 0.306 mmol, 2.5 mol%) and LiHMDS (1 M in THF, 17.1 mL, 17.1 mmol, 1.40 equiv.) were added to a solution of *tert*-butyl 4-(2-chloro-5-ethylpyrimidin-4-yl)piperazine-1-carboxylate (**6**, 4.00 g, 12.2 mmol, 1.00 equiv.) in dry 1,4-dioxane (150 mL). The mixture was heated to 50 °C and stirred for 1 h. The reaction was quenched by the addition of sat. $NaHCO_3$ (200 mL) at room temperature. The mixture was extracted with DCM (5 x 50 mL) and the combined extracts were dried over Na_2SO_4 and concentrated. The residue was purified by column chromatography (120 g silica gel, gradient 1–5% MeOH/DCM, 10 CV) to afford the product *tert*-butyl 4-(2-amino-5-ethylpyrimidin-4-yl)piperazine-1-carboxylate (**7**, 3.78 g, quant.). ^{1}H-NMR (400 MHz, $CDCl_3$): δ [ppm] = 7.84 (s, 1H), 4.77 (s, 2H), 3.51 (dd, J = 6.3, 4.0 Hz, 4H), 3.32 (dd, J = 6.2, 4.0 Hz, 4H), 2.47 (q, J = 7.5 Hz,

2H), 1.47 (s, 10H), 1.20 (t, J = 7.5 Hz, 3H). ^{13}C-NMR (101 MHz, $CDCl_3$): δ [ppm] = 165.7, 160.7, 160.6, 157.0, 154.9, 114.4, 80.1, 48.0, 28.5, 22.8, 14.0. ESI(+) calcd. for $[M+H]^+$ **7**: *m*/*z* = 308.2; found: 308.4.

*2.4. Step iv: 5-Ethyl-4-(piperazin-1-yl)pyrimidin-2-amine (**8**)*

tert-Butyl 4-(2-amino-5-ethylpyrimidin-4-yl)piperazine-1-carboxylate (**7**, 0.40 g, 1.30 mmol, 1.00 equiv.) was dissolved in 1,4-dioxane (10 mL), HCl (4 M in 1,4-dioxane, 6.50 mL, 26.0 mmol, 20.0 equiv.) was added, and the mixture was stirred for 1 h at reflux. After cooling to room temperature, the suspension was filtered, and the precipitate was washed with 1,4-dioxane (5 mL). The precipitate was dissolved in water (5 mL) and the solution was basified with 10 M NaOH. The aq. layer was extracted with DCM (10 x 20 mL) and the combined organic extracts were dried over Na_2SO_4 and concentrated. 5-Ethyl-4-(piperazin-1-yl)pyrimidin-2-amine (**8**, 0.269 g, quant.) was used without further purification. ^{1}H-NMR (400 MHz, $CDCl_3$): δ [ppm] = 7.82 (s, 1H), 4.69 (s, 2H), 3.33–3.28 (m, 4H), 2.98–2.91 (m, 4H), 2.46 (q, J = 7.5 Hz, 2H), 2.00 (s, 1H), 1.19 (t, J = 7.5 Hz, 3H). ^{13}C-NMR (101 MHz, $CDCl_3$): δ [ppm] = 165.9, 161.1, 157.7, 114.5, 49.5, 46.2, 22.9, 14.0. ESI(+) calcd. for $[M+H]^+$ **8**: *m*/*z* = 208.1; found: 208.2.

*2.5. Step v: 2-(Chloromethyl)-1-(methoxymethyl)-6-(trifluoromethyl)-1H-benzo[d]imidazole (**10**)*

MOMBr (1.01 mL, 12.8 mmol, 1.35 equiv.) was slowly added at −40 °C to a solution of 2-(chloromethyl)-5-(trifluoromethyl)-1*H*-benzo[*d*]imidazole (**9**, 2.22 g, 9.46 mmol, 1.00 equiv.) and DIPEA (5.79 mL, 33.1 mmol, 3.50 equiv.) in dry THF (75 mL). The reaction was stirred for 17 h at this temperature and then quenched with water (50 mL) at room temperature. The aq. layer was extracted with DCM (3 x 100 mL) and the combined organic extracts were dried over Na_2SO_4 and concentrated. The product was obtained as a mixture of regioisomers, which were separated by column chromatography (40 g silica gel, gradient 0–1% MeOH/DCM, 20 CV) to afford 2-(chloromethyl)-1-(methoxymethyl)-6-(trifluoromethyl)-1*H*-benzo[*d*]imidazole (**10**) and 2-(chloromethyl)-1-(methoxymethyl)-5-(trifluoromethyl)-1*H*-benzo[*d*]imidazole (1.78 g, 68% overall yield, 10:8.7 ratio). Only the major isomer **10** (as depicted in Scheme S1) was used for further reactions and characterization. ^{1}H-NMR (400 MHz, $CDCl_3$): δ [ppm] = 7.86 (d, J = 8.4 Hz, 1H), 7.79 (s, 1H), 7.58 (d, J = 8.4 Hz, 1H), 5.65 (s, 2H), 4.91 (s, 2H), 3.37 (s, 3H). ^{13}C-NMR (101 MHz, $CDCl_3$): δ [ppm] = 152.0, 144.1, 135.1, 126.7 (q, *J* = 32.2 Hz), 124.6 (q, *J* = 271.6 Hz), 121.0, 120.4, 108.2, 75.3, 57.0, 36.4. ESI(+) calcd. for $[M+H]^+$ **10**: *m*/*z* = 279.0; found: 279.0.

*2.6. Step vi: 5-Ethyl-4-(4-((1-(methoxymethyl)-6-(trifluoromethyl)-1H-benzo[d]imidazol-2-yl)methyl)piperazin-1-yl)pyrimidin-2-amine (**12**)*

2-(Chloromethyl)-1-(methoxymethyl)-6-(trifluoromethyl)-1*H*-benzo[*d*]imidazole (**10**, 0.269 g, 0.965 mmol, 1.00 equiv.) was dissolved in dry acetone (15 mL) and NaI (0.144 g, 0.965 mmol, 1.00 equiv.) was added at room temperature. The mixture was stirred for 30 min, then 5-ethyl-4-(piperazin-1-yl)pyrimidin-2-amine (**8**, 0.200 g, 0.965 mmol, 1.00 equiv.) was added at 0 °C, followed by DIPEA (421 μL, 2.41 mmol, 2.50 equiv.). The reaction was allowed to warm up to room temperature over 17 h. The reaction was diluted with DCM (40 mL) and water (10 mL). The aq. layer was extracted with DCM (3 x 40 mL) and the combined organic extracts were dried over Na_2SO_4 and concentrated. The residue was purified by column chromatography (80 g silica gel, gradient 0.5–3% MeOH/DCM, 10 CV) to afford the product 5-ethyl-4-(4-((1-(methoxymethyl)-6-(trifluoromethyl)-1*H*-benzo[*d*]imidazol-2-yl)methyl)piperazin-1-yl)pyrimidin-2-amine (**12**, 0.347 g, 80% yield). ^{1}H-NMR (400 MHz, $CDCl_3$): δ [ppm] = 7.82 (d, J = 8.5 Hz, 1H), 7.79 (s, 1H), 7.74 (s, 1H), 7.54 (d, J = 8.5 Hz, 1H), 5.74 (s, 2H), 5.15 (s, 2H), 3.94 (s, 2H), 3.51–3.40 (m, 4H), 3.36 (s, 3H), 2.71–2.59 (m, 4H), 2.46 (q, J = 7.4 Hz, 2H), 1.18 (t, *J* = 7.4 Hz, 3H). ^{13}C-NMR (101 MHz, $CDCl_3$): δ [ppm] = 165.2, 159.2, 153.8, 153.6, 144.6, 135.4, 125.8 (q, J = 32.3 Hz), 124.8 (q, J = 272.0 Hz), 120.4, 119.8 (q, J = 3.4 Hz), 114.1, 108.0 (q, J = 4.1 Hz), 75.2, 56.8, 55.7, 53.4, 47.9, 23.2, 13.9. ESI(+) calcd. for $[M+H]^+$ **12**: *m*/*z* = 450.2; found: 450.4.

*2.7. Step vii: tert-Butyl (5-ethyl-4-(4-((1-(methoxymethyl)-6-(trifluoromethyl)-1H-benzo[d]imidazol-2-yl)methyl)piperazin-1-yl)pyrimidin-2-yl)carbamate (**13**)*

5-Ethyl-4-(4-((1-(methoxymethyl)-6-(trifluoromethyl)-1*H*-benzo[*d*]imidazol-2-yl)methyl)piperazin-1-yl)pyrimidin-2-amine (**12**, 0.560 g, 1.25 mmol, 1.00 equiv.) was dissolved in a minimal amount of dry *t*BuOH (2 mL) and stirred at 40 °C, while Boc_2O (315 µL, 1.37 mmol, 1.10 equiv.) was added. After stirring for 17 h, the suspension was concentrated and purified by column chromatography (80 g silica gel, gradient 0–5% MeOH/DCM, 10 CV) to afford the product *tert*-butyl (5-ethyl-4-(4-((1-(methoxymethyl)-6-(trifluoromethyl)-1*H*-benzo[*d*]imidazol-2-yl)methyl)piperazin-1-yl)pyrimidin-2-yl)carbamate (**13**, 0.685 g, quant.). ^{1}H-NMR (400 MHz, $CDCl_3$): δ [ppm] = 8.06 (s, 1H), 7.81 (d, J = 8.4 Hz, 1H), 7.78 (s, 1H), 7.57 (s, 1H), 7.53 (dd, J = 8.5, 1.3 Hz, 1H), 5.73 (s, 2H), 3.93 (s, 2H), 3.47–3.40 (m, 4H), 3.35 (s, 3H), 2.68–2.61 (m, 4H), 2.50 (q, J = 7.5 Hz, 2H), 1.50 (s, 9H), 1.18 (t, J = 7.5 Hz, 3H). ^{13}C-NMR (101 MHz, $CDCl_3$): δ [ppm] = 164.7, 157.9, 155.3, 153.7, 150.9, 144.6, 135.4, 125.8 (q, J = 32.3 Hz), 124.8 (q, J = 271.9 Hz), 120.4, 119.7 (q, J = 3.5 Hz), 117.9, 107.9 (q, J = 4.2 Hz), 80.8, 75.2, 56.7, 55.7, 53.4, 47.8, 28.4, 23.3, 14.0. ESI(+) calcd. for $[M+H]^+$ **13**: *m*/*z* = 550.3; found: 550.6.

*2.8. Step viii: tert-Butyl (6-chloro-3-nitropyridin-2-yl)(5-ethyl-4-(4-((1-(methoxymethyl)-6-(trifluoromethyl)-1H-benzo[d]imidazol-2-yl)methyl)piperazin-1-yl)pyrimidin-2-yl)carbamate (**15**)*

(5-Ethyl-4-(4-((1-(methoxymethyl)-6-(trifluoromethyl)-1*H*-benzo[*d*]imidazol-2-yl)methyl)piperazin-1-yl)pyrimidin-2-yl)carbamate (**13**, 47.5 mg, 86.4 µmol, 1.00 equiv.), 2-bromo-6-chloro-3-nitropyridine (**14**, 41.0 mg, 173 µmol, 2.00 equiv.), Cs_2CO_3 (113 mg, 345 µmol, 4.00 equiv.), and XantPhos Pd G4 (4.2 mg, 5mol%) were put in a flask and thoroughly flushed with argon. Dry toluene (5 mL) was added, and the mixture was stirred at 55 °C for 3 d. The mixture was diluted with DCM (15 mL) and water (10 mL). The aq. layer was extracted with DCM (3 x 15 mL) and the combined organic extracts were dried over Na_2SO_4 and concentrated. The residue was purified by column chromatography (40 g silica gel, gradient 0–2.5% MeOH/DCM, 10 CV) to afford the product *tert*-butyl (6-chloro-3-nitropyridin-2-yl)(5-ethyl-4-(4-((1-(methoxymethyl)-6-(trifluoromethyl)-1*H*-benzo[*d*]imidazol-2-yl)methyl)piperazin-1-yl)pyrimidin-2-yl)carbamate (**15**, 42.2 mg, 69% yield). ^{1}H-NMR (400 MHz, $CDCl_3$): δ [ppm] = 8.36 (d, J = 8.4 Hz, 1H), 8.11 (s, 1H), 7.83 (d, J = 8.4 Hz, 1H), 7.78 (s, 1H), 7.54 (dd, J = 8.5, 1.2 Hz, 1H), 7.38 (d, J = 8.4 Hz, 1H), 5.72 (s, 2H), 3.91 (s, 2H), 3.35 (s, 3H), 3.33–3.28 (m, 4H), 2.60–2.56 (m, 4H), 2.53 (q, J = 7.6 Hz, 2H), 1.43 (s, 9H), 1.22 (t, J = 7.5 Hz, 3H). ^{13}C-NMR (101 MHz, $CDCl_3$): δ [ppm] = 164.6, 157.9, 156.4, 153.7, 153.5, 150.9, 148.0, 144.5, 140.8, 136.4, 135.4, 124.8 (q, J = 272.1 Hz), 125.8 (q, J = 32.3 Hz), 123.1, 120.4, 120.2, 119.8 (q, J = 3.4 Hz), 107.9 (q, J = 4.1 Hz), 83.8, 75.2, 56.8, 55.7, 53.2, 47.7, 28.0, 23.2, 13.5. ESI(+) calcd. for $[M+Na]^+$ **15**: *m*/*z* = 728.2; found: 728.4.

*2.9. Step ix: N-(6-Chloro-3-nitropyridin-2-yl)-5-ethyl-4-(4-((6-(trifluoromethyl)-1H-benzo[d]imidazol-2-yl)methyl)piperazin-1-yl)pyrimidin-2-amine (**2**)*

HCl (4 M in 1,4-dioxane, 0.300 mL, 1.20 mmol, 20.0 equiv.) was added to a solution of *tert*-butyl (6-chloro-3-nitropyridin-2-yl)(5-ethyl-4-(4-((1-(methoxymethyl)-6-(trifluoromethyl)-1*H*-benzo[*d*]imidazol-2-yl)methyl)piperazin-1-yl)pyrimidin-2-yl)carbamate (**15**, 42.4 mg, 60.0 µmol, 1.00 equiv.) in dry 1,4-dioxane (6 mL). The reaction was stirred at reflux for 1 h, diluted with DCM (40 mL), and basified with 1 M NaOH (10 mL). The aq. layer was extracted with DCM (3 x 20 mL) and the combined extracts were dried over Na_2SO_4 and concentrated. The residue was purified by column chromatography (40 g silica gel, gradient 0–5% MeOH/DCM, 10 CV) to afford the product *N*-(6-chloro-3-nitropyridin-2-yl)-5-ethyl-4-(4-((6-(trifluoromethyl)-1*H*-benzo[*d*]imidazol-2-yl)methyl)piperazin-1-yl)pyrimidin-2-amine (**2**, 29.6 mg, 88% yield). ^{1}H-NMR (400 MHz, $CDCl_3$): δ [ppm] = 10.19 (br, 1H), 9.96 (br, 1H), 8.45 (d, J = 8.6 Hz, 1H), 8.10 (s, 1H), 8.03–7.55 (m, 2H), 7.54–7.49 (m, 1H), 6.95 (d, J = 8.6 Hz, 1H), 3.93 (s, 2H), 3.65–3.57 (m, 4H), 2.77– 2.68 (m, 4H), 2.57 (q, J = 7.5 Hz, 2H), 1.25 (t, J = 7.5 Hz, 3H). ^{1}H-NMR (600 MHz, DMSO-d_6): δ [ppm] = 12.79 (s, 1H), 10.47 (s, 1H), 8.42 (d, J = 8.5 Hz, 1H), 7.98 (s, 1H), 7.89 (br, 1H), 7.68 (br, 1H), 7.52–7.44 (m, 1H), 7.25 (d, J = 8.5 Hz, 1H), 3.84 (s, 2H), 2.62–2.54 (m, 4H), 2.53–2.50 (m, 2H), 1.14 (t, J = 7.5 Hz, 3H). ^{13}C-NMR (151 MHz, DMSO-

d6): δ [ppm] = 163.6, 157.3, 155.3, 154.4 (br), 151.9, 146.4, 142.5 (br), 137.9, 136.9 (br), 134.4, 125.1 (q, J = 271.8 Hz), 122.2 (br), 118.5 (br), 117.30, 117.27, 115.7 (br), 112.2 (br), 108.8 (br), 55.5, 52.7, 47.0, 22.6, 13.5. HRMS: ESI(+) calcd. for **2** ($C_{24}H_{23}ClF_3N_9O_2$) $[M+H]^+$: m/z = 562.16881; found: 562,16871; rel. deviation 0.17 ppm. HPLC: retention time: 17.96 min; purity: 98.16% (254 nm), 98.26% (230 nm).

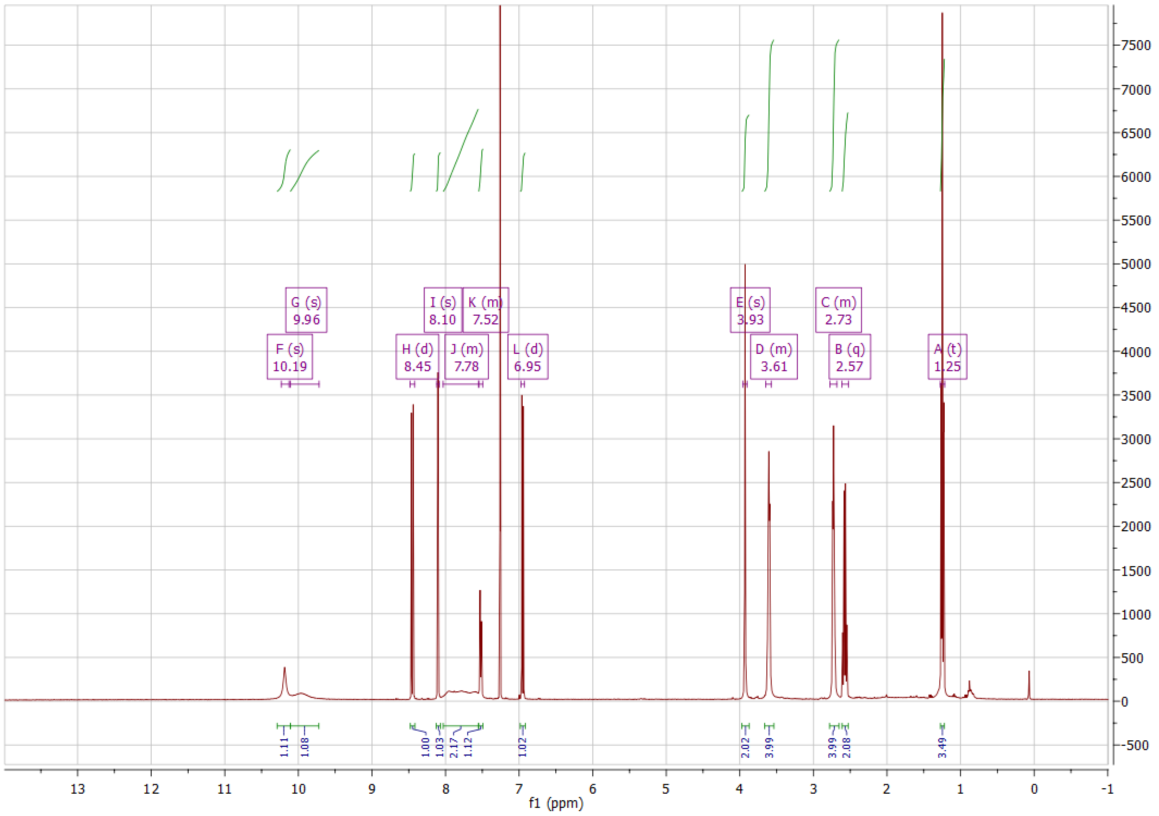

Figure S3. ^{1}H-NMR spectrum of **2** in $CDCl_3$ (note: the benzimidazole tautomerism led to the broadening of some aromatic signals).

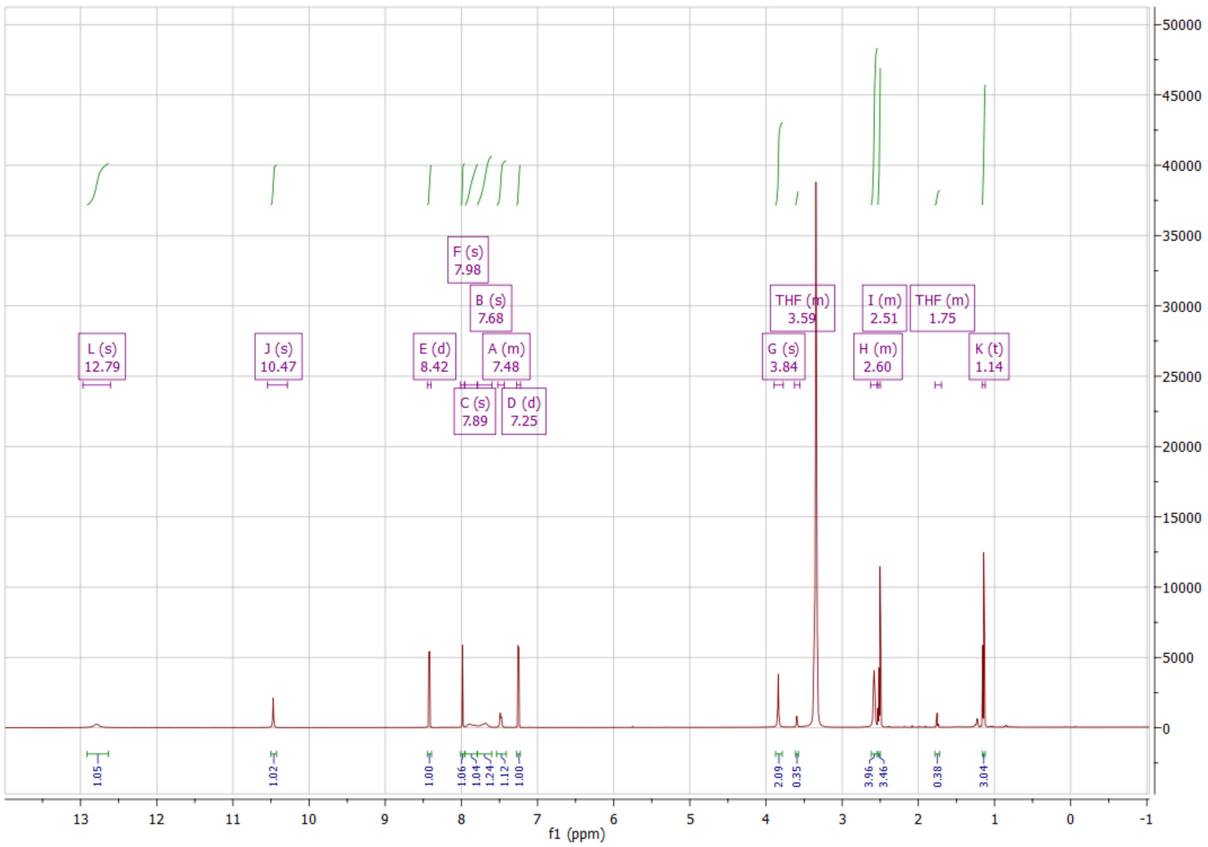

Figure S4. ^{1}H-NMR spectrum of **2** in DMSO-d$_6$ (note: the benzimidazole tautomerism led to the broadening of some aromatic signals; two CH_2-groups of the piperazine are overlapped by the water signal; the CH_2-group adjacent to CH_3 is partially overlapped by the residual DMSO signal).

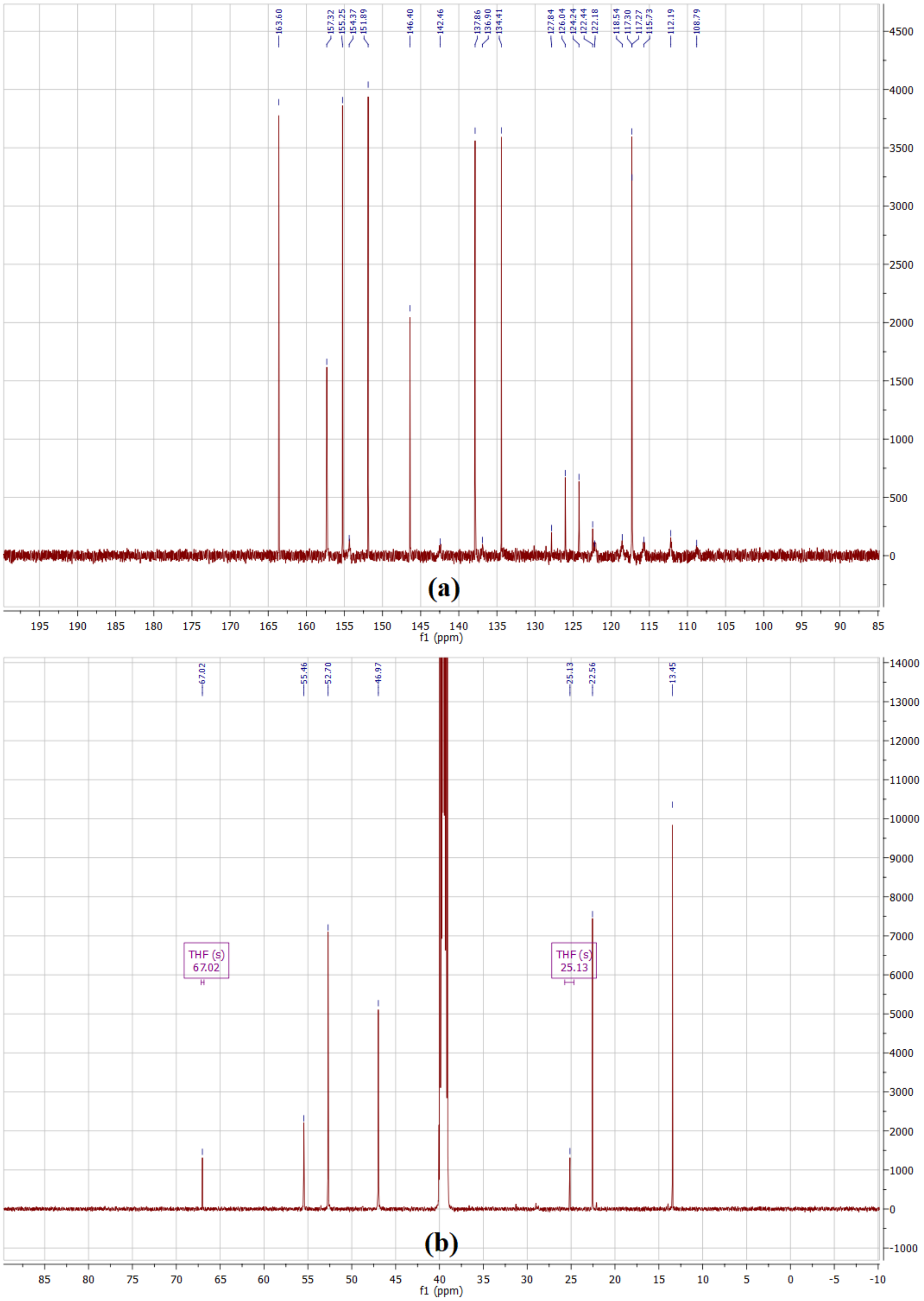

Figure S5. ^{13}C-NMR spectrum of **2** in DMSO-d_6: (**a**) region from 200 ppm to 85 ppm; (**b**) region from 90 ppm to −10 ppm (note: the benzimidazole tautomerism led to the signal broadening and splitting of benzimidazole signals).

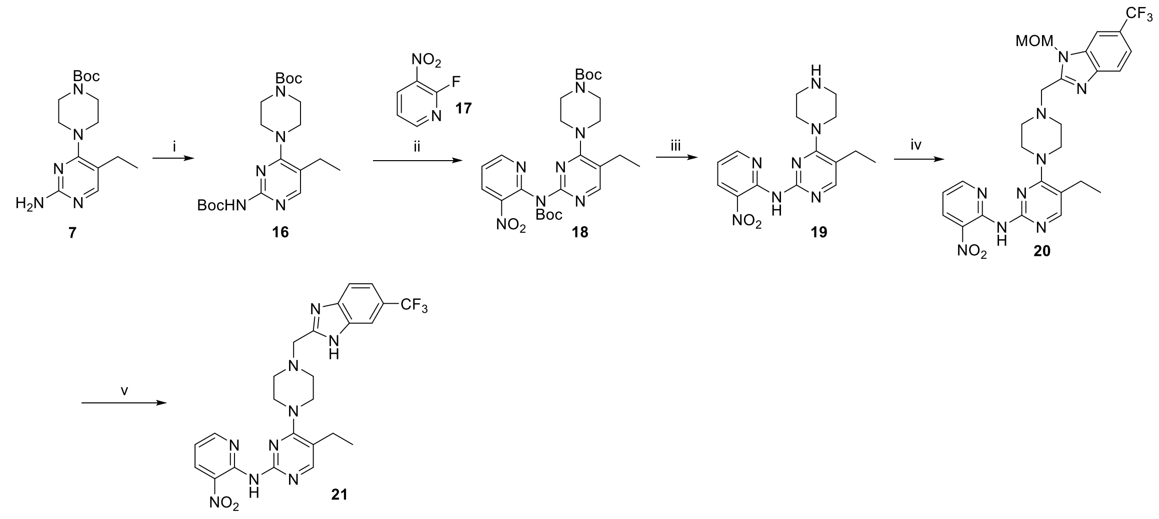

Scheme S2. Synthesis of the unreactive analog **21.**

*2.10. Step i: tert-Butyl 4-(2-((tert-butoxycarbonyl)amino)-5-ethylpyrimidin-4-yl)piperazine-1-carboxylate (**16**)*

tert-Butyl 4-(2-amino-5-ethylpyrimidin-4-yl)piperazine-1-carboxylate (**7**, 0.100 g, 0.325 mmol, 1.00 equiv.) was dissolved in a minimal amount of dry *t*BuOH (2 mL) and stirred at 40 °C, while Boc_2O (82.2 µL, 0.358 mmol, 1.10 equiv.) was added. The reaction was stirred for 2 h and hexane (10 mL) was added. The precipitate was filtered off and washed with hexane. Drying in vacuo afforded the product *tert*-butyl 4-(2-((*tert*-butoxycarbonyl)amino)-5-ethylpyrimidin-4-yl)piperazine-1-carboxylate (**16**, 0.106 g, 80% yield). ^{1}H-NMR (400 MHz, $CDCl_3$): δ [ppm] = 8.08 (s, 1H), 7.55 (s, 1H), 3.52 (dd, J = 6.4, 3.7 Hz, 4H), 3.40 (dd, J = 6.4, 3.8 Hz, 4H), 2.53 (q, J = 7.5 Hz, 2H), 1.51 (s, 9H), 1.47 (s, 9H), 1.21 (t, J = 7.5 Hz, 3H). ^{13}C-NMR (101 MHz, $CDCl_3$): δ [ppm] = 164.9, 157.5, 155.0, 154.9, 150.8, 117.9, 81.0, 80.2, 47.9, 28.5, 28.4, 23.2, 13.8. ESI(+) calcd. for $[M+H]^+$ **16**: *m*/*z* = 408.3; found: 408.4.

*2.11. Step ii: tert-Butyl 4-(2-((tert-butoxycarbonyl)(3-nitropyridin-2-yl)amino)-5-ethylpyrimidin-4-yl)piperazine-1-carboxylate (**18**)*

A solution of *tert*-butyl 4-(2-((*tert*-butoxycarbonyl)(3-nitropyridin-2-yl)amino)-5-ethylpyrimidin-4-yl)piperazine-1-carboxylate (**16**, 0.235 g, 0.578 mmol, 1.00 equiv.) in dry DMF (10 mL) was cooled to 0 °C, and NaH (60% dispersion in mineral oil, 30.0 mg, 0.750 mmol, 1.30 equiv.) was added. The mixture was stirred at room temperature for 1 h, and then 2-fluoro-3-nitropyridine (**17**, 228 µL, 2.31 mmol, 4.00 equiv.) was added. The reaction was stirred for 7 d at room temperature and quenched with water (20 mL). The aq. layer was extracted with DCM (3 × 40 mL) and the combined organic extracts were dried over Na_2SO_4 and concentrated. The residue was purified by column chromatography (80 g silica gel, gradient 2.5% MeOH/DCM, 10 CV, then again 70–100% EtOAc/hexane 10 CV) to afford the product 4-(2-((*tert*-butoxycarbonyl)(3-nitropyridin-2-yl)amino)-5-ethylpyrimidin-4-yl)piperazine-1-carboxylate (**18**, 0.190 g, 62% yield). ^{1}H-NMR (400 MHz, $CDCl_3$): δ [ppm] = 8.67 (dd, J = 4.7, 1.7 Hz, 1H), 8.44 (dd, J = 8.1, 1.7 Hz, 1H), 8.18 (s, 1H), 7.43 (dd, J = 8.1, 4.7 Hz, 1H), 3.41 (dd, J = 6.3, 3.9 Hz, 4H), 3.27 (dd, J = 6.3, 3.9 Hz, 4H), 2.55 (q, J = 7.6 Hz, 2H), 1.46 (s, 9H), 1.43 (s, 9H), 1.24 (t, J = 7.5 Hz, 3H). ^{13}C-NMR (101 MHz, $CDCl_3$): δ [ppm] = 164.6, 157.3, 154.9, 152.7, 151.1, 147.8, 142.4, 134.1, 122.8, 120.0, 83.7, 80.2, 47.8, 28.6, 28.0, 23.2, 13.5. ESI(+) calcd. for $[M+H]^+$ **18**: *m*/*z* = 530.3; found: 530.3.

*2.12. Step iii: 5-Ethyl-N-(3-nitropyridin-2-yl)-4-(piperazin-1-yl)pyrimidin-2-amine (**19**)*

HCl (4 M in 1,4-dioxane, 1.70 mL, 6.80 mmol, 20.0 equiv.) was added to a solution of 4-(2-((*tert*-butoxycarbonyl)(3-nitropyridin-2-yl)amino)-5-ethylpyrimidin-4-yl)piperazine-1-carboxylate (**18**, 0.180 g, 0.340 mmol, 1.00 equiv.) in 1,4-dioxane (5 mL). The mixture was stirred at 50 °C for 4 h, diluted with DCM (20 mL), and basified with 1 M NaOH (10 mL). The aq. layer was extracted with

DCM (5 × 20 mL) and the combined organic extracts were dried over Na_2SO_4 and concentrated. The residue was purified by column chromatography (80 g silica gel, gradient 0.5–5% MeOH/DCM, 10 CV) to afford the product 5-ethyl-*N*-(3-nitropyridin-2-yl)-4-(piperazin-1-yl)pyrimidin-2-amine (**19**, 0.100 g, 89% yield). ^{1}H-NMR (400 MHz, $CDCl_3$): δ [ppm] = 9.99 (s, 1H), 8.66 (dd, J = 4.6, 1.8 Hz, 1H), 8.50 (dd, J = 8.3, 1.8 Hz, 1H), 8.15 (s, 1H), 6.99 (dd, J = 8.3, 4.6 Hz, 1H), 3.46–3.41 (m, 4H), 3.01–2.93 (m, 4H), 2.57 (q, J = 7.5 Hz, 2H), 1.25 (t, J = 7.5 Hz, 3H). ^{13}C-NMR (101 MHz, $CDCl_3$): δ [ppm] = 165.2, 157.7, 155.5, 154.8, 148.2, 135.2, 131.6, 118.7, 116.1, 49.4, 46.2, 23.4, 13.8. ESI(+) calcd. for $[M+H]^+$ **19**: *m*/*z* = 330.2; found: 330.4.

*2.13. Step iv: 5-Ethyl-4-(4-((1-(methoxymethyl)-6-(trifluoromethyl)-1H-benzo[d]imidazol-2-yl)methyl)piperazin-1-yl)-N-(3-nitropyridin-2-yl)pyrimidin-2-amine (**20**)*

The same method as for compound **8** was employed to 5-ethyl-*N*-(3-nitropyridin-2-yl)-4-(piperazin-1-yl)pyrimidin-2-amine (**19**, 60.0 mg) to afford the product 5-ethyl-4-(4-((1-(methoxymethyl)-6-(trifluoromethyl)-1*H*-benzo[*d*]imidazol-2-yl)methyl)piperazin-1-yl)-*N*-(3-nitropyridin-2-yl)pyrimidin-2-amine (**20**, 71.0 mg, 68% yield). ^{1}H-NMR (400 MHz, $CDCl_3$): δ [ppm] = 9.99 (s, 1H), 8.64 (dd, J = 4.4, 1.4 Hz, 1H), 8.48 (dd, J = 8.3, 1.7 Hz, 1H), 8.16 (s, 1H), 7.82 (d, J = 8.4 Hz, 1H), 7.79 (s, 1H), 7.53 (dd, J = 8.4, 1.0 Hz, 1H), 6.99 (dd, J = 8.3, 4.6 Hz, 1H), 5.75 (s, 2H), 3.94 (s, 2H), 3.50–3.40 (m, 4H), 3.36 (s, 3H), 2.71–2.63 (m, 4H), 2.55 (q, J = 7.5 Hz, 2H), 1.23 (t, J = 7.5 Hz, 3H). ^{13}C-NMR (101 MHz, $CDCl_3$): δ [ppm] = 164.9, 157.5, 155.3, 154.7, 153.7, 145.0, 144.5, 135.4, 135.1, 131.8, 125.8 (q, J = 32.3 Hz), 124.8 (q, J = 271.9 Hz), 120.4, 119.8 (q, J = 3.4 Hz), 118.7, 116.3, 108.0 (q, J = 4.3 Hz), 75.2, 56.8, 55.7, 53.3, 47.8, 23.3, 13.7. ESI(+) calcd. for $[M+H]^+$ **20**: *m*/*z* = 572.2; found: 572.4.

*2.14. Step v: 5-Ethyl-N-(3-nitropyridin-2-yl)-4-(4-((6-(trifluoromethyl)-1H-benzo[d]imidazol-2-yl)methyl)piperazin-1-yl)pyrimidin-2-amine (**21**)*

HCl (4 M in 1,4-dioxane, 0.766 mL, 3.07 mmol, 40.0 equiv.) was added to a solution of 5-ethyl-4-(4-((1-(methoxymethyl)-6-(trifluoromethyl)-1*H*-benzo[*d*]imidazol-2-yl)methyl)piperazin-1-yl)-*N*-(3-nitropyridin-2-yl)pyrimidin-2-amine (**20**, 43.8 mg, 77.0 µmol, 1.00 equiv.) in dry 1,4-dioxane (4 mL). The reaction was stirred at 50 °C for 4 h, diluted with DCM (40 mL), and basified with 1 M NaOH (10 mL). The aq. layer was extracted with DCM (3 × 20 mL) and the combined organic extracts were dried over Na_2SO_4 and concentrated. The residue was purified by column chromatography (40 g silica gel, gradient 0.5–10% MeOH/DCM, 10 CV) to afford the product 5-ethyl-*N*-(3-nitropyridin-2-yl)-4-(4-((6-(trifluoromethyl)-1*H*-benzo[*d*]imidazol-2-yl)methyl)piperazin-1-yl)pyrimidin-2-amine (**21**, 32.0 mg, 80% yield). (Note: the product appears as a 50:50 mixture of tautomers in the ^{1}H-NMR due to different protonation sites of the benzimidazole with overlapping and distinct signals denoted as H_a and H_b without making a specific assignment). ^{1}H-NMR (400 MHz, $CDCl_3$): δ [ppm] = 10.31 (s, $1H_{a/b}$), 10.23 (s, $1H_{a/b}$), 10.01 (s, 1H), 8.61 (dd, J = 4.6, 1.8 Hz, 1H), 8.50 (dd, J = 8.3, 1.8 Hz, 1H), 8.15 (s, 1H), 8.00 (s, $1H_{a/b}$), 7.80 (d, J = 8.4 Hz, $1H_a$), 7.76 (s, $1H_{a/b}$), 7.55 (d, J = 8.3 Hz, $1H_a$), 7.52 (d, J = 8.6 Hz, $1H_b$), 7.51 (d, J = 8.6 Hz, $1H_b$), 7.00 (dd, J = 8.3, 4.6 Hz, 1H), 3.90 (s, 2H), 3.50–3.41 (m, 4H), 2.73–2.63 (m, 4H), 2.55 (q, J = 7.5 Hz, 2H), 1.23 (t, J = 7.5 Hz, 3H). ^{1}H-NMR (400 MHz, DMSO-d_6): δ [ppm] = 12.79 (s, 1H), 10.16 (s, 1H), 8.56 (dd, J = 4.6, 1.7 Hz, 1H), 8.36 (dd, J = 8.1, 1.6 Hz, 1H), 7.94 (s, 1H), 7.87 (br, 1H), 7.69 (br, 1H), 7.52–7.43 (m, 1H), 7.22 (dd, J = 8.1, 4.6 Hz, 1H), 3.82 (s, 2H), 3.34–3.28 (m, 4H), 2.59–2.53 (m, 4H), 2.50–2.45 (m, 2H), 1.13 (t, J = 7.4 Hz, 3H). ^{13}C-NMR (101 MHz, DMSO-d_6): δ [ppm] = 163.90, 157.2, 156.0, 154.5 (br), 152.5, 146.1, 142.5 (br), 136.5, 134.3, 125.2 (q, J = 271.6 Hz), 122.3 (br), 118.5 (br), 118.0, 116.7, 115.8 (br), 112.3 (br), 108.5 (br), 55.5, 52.7, 47.1, 22.4, 13.5. HRMS: ESI(+) calcd. for **21** ($C_{24}H_{24}F_3N_9O_2$) $[M+H]^+$: *m*/*z* = 528.20778; found: 528,20794; rel. deviation 0.30 ppm. HPLC: retention time: 13.96 min; purity: 98.36% (254 nm), 97.70% (230 nm).

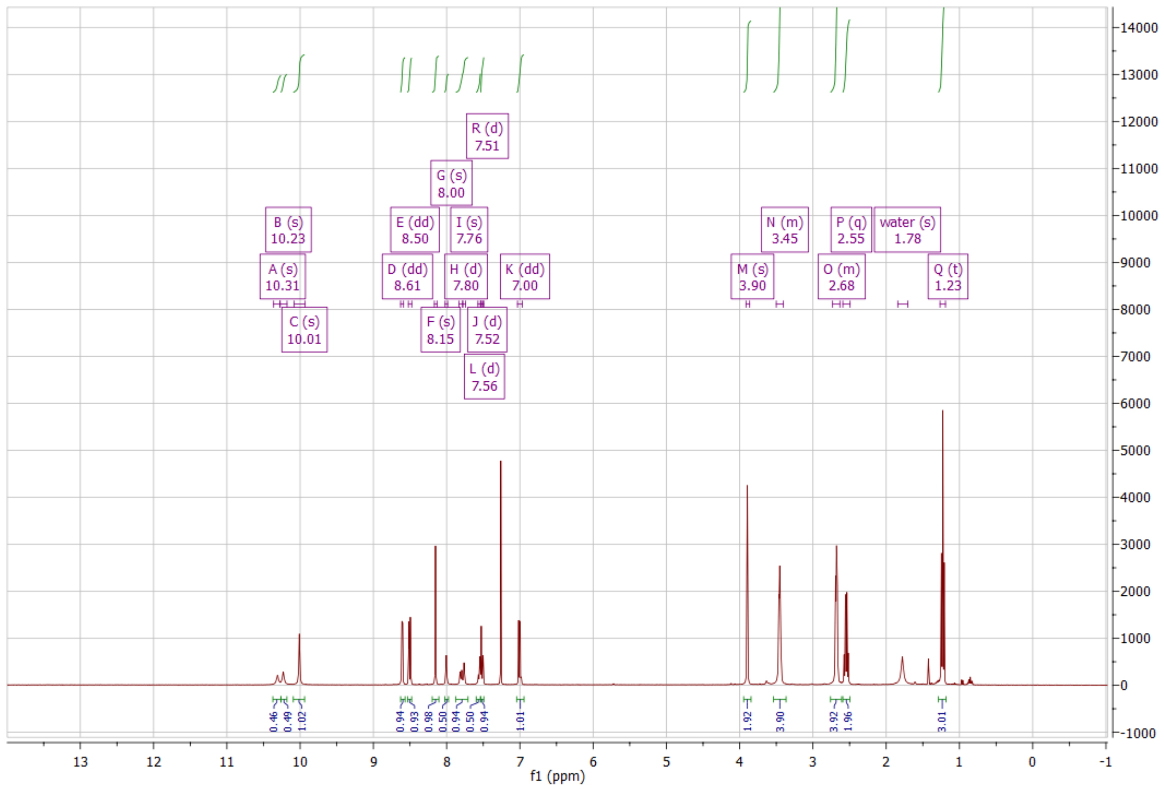

Figure S6. ^{1}H-NMR spectrum of **21** in $CDCl_3$ (note: the product appears as a 50:50 mixture of benzimidazole tautomers with some overlapping signals).

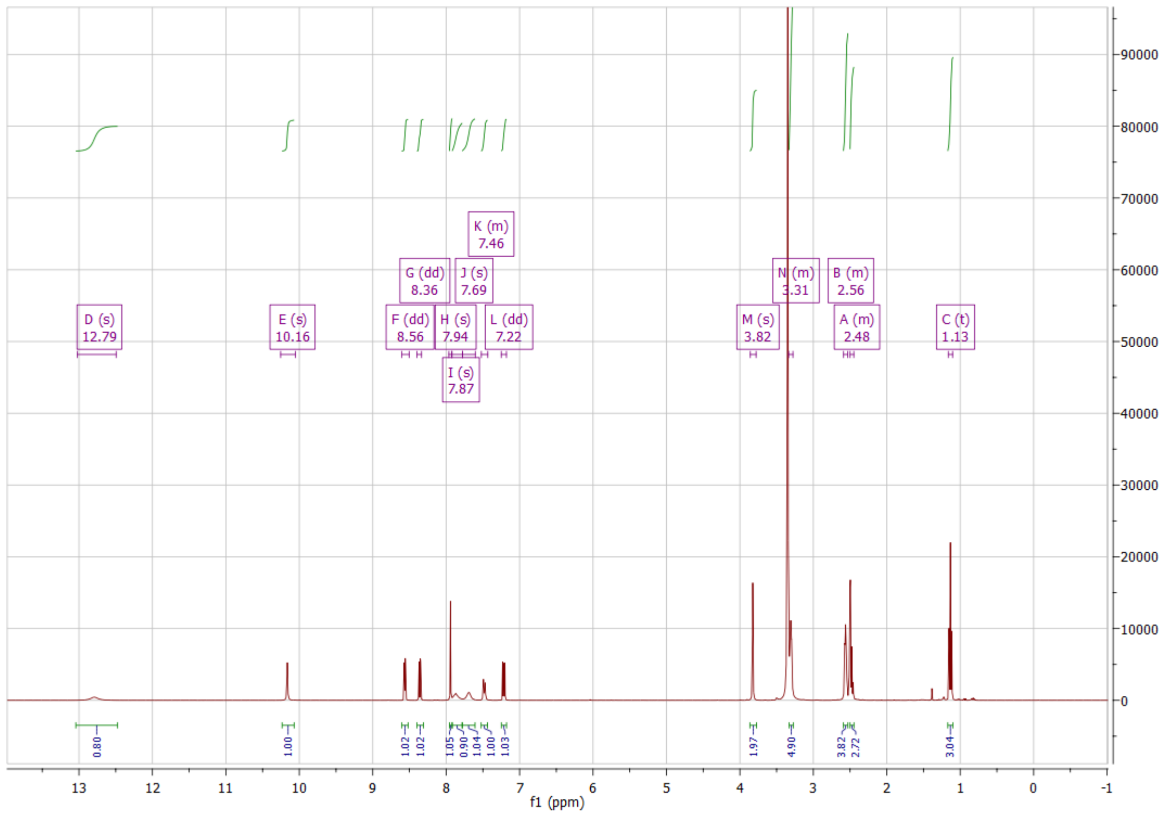

Figure S7. ^{1}H-NMR spectrum of **21** in DMSO-d_6 (note: the benzimidazole tautomerism led to the broadening of some aromatic signals; two CH_2-groups of the piperazine are partially overlapped by the water signal; the CH_2-group adjacent to CH_3 is partially overlapped by the residual DMSO signal).

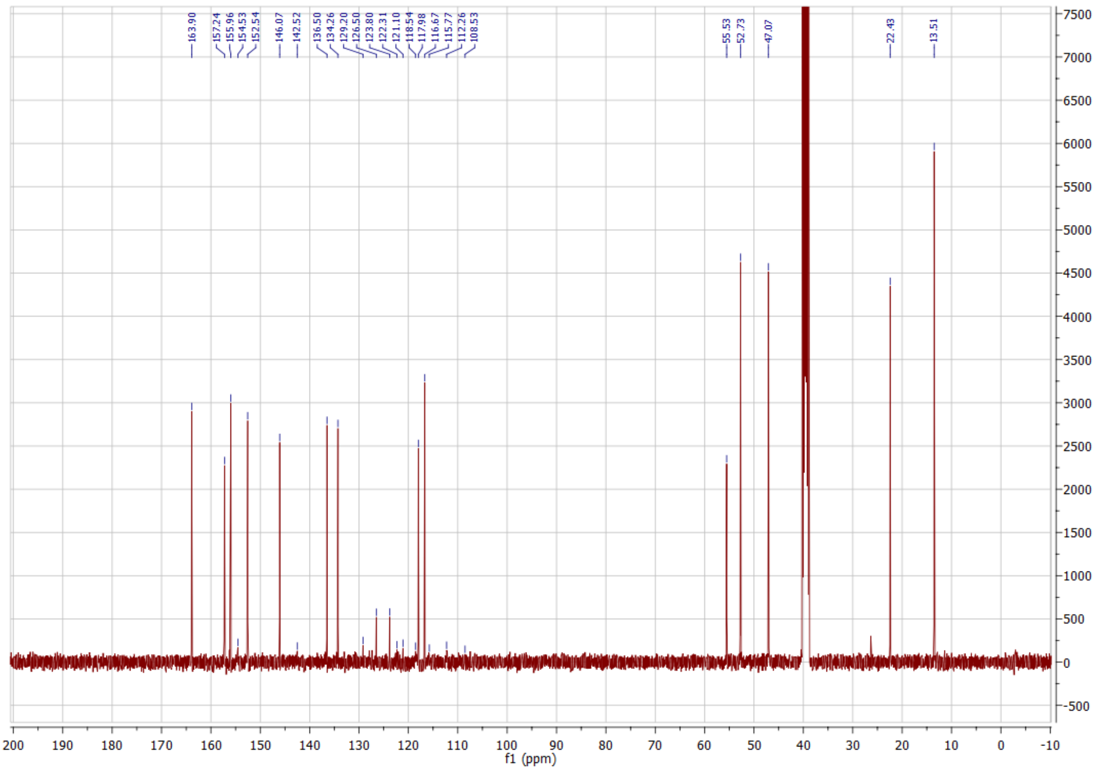

Figure S8. ^{13}C-NMR spectrum of 21 in DMSO-d_6. (note: the benzimidazole tautomerism led to signal broadening and splitting of benzimidazole signals).

3. Glutathione (GSH) Stability Assay

HPLC specifications: Column: Phenomenex Kinetex 2.6 µM C8 100 Å 150 × 4.6 mm
Injection volume: 5 µL flow rate: 0.5 mL/min at 23 °C
HPLC-run **A** for **2**:

- 0 min: 40% MeOH, 60% phosphate buffer pH 2.3;
- 15 min: 85% MeOH, 15% phosphate buffer pH 2.3;
- 20 min: 85% MeOH, 15% phosphate buffer pH 2.3;
- 22 min: 40% MeOH, 60% phosphate buffer pH 2.3;
- 28 min: 40% MeOH, 60% phosphate buffer pH 2.3.

HPLC-run **B** for Afatinib:

- 0 min: 40% MeOH, 60% phosphate buffer pH 2.3;
- 9 min: 95% MeOH, 5% phosphate buffer pH 2.3;
- 10 min: 95% MeOH, 5% phosphate buffer pH 2.3;
- 11 min: 40% MeOH, 60% phosphate buffer pH 2.3;
- 16 min: 40% MeOH, 60% phosphate buffer pH 2.3.

The half-lives $t_{1/2}$ for title compound **2** and Afatinib were calculated to be 55 h and 5 h, respectively.

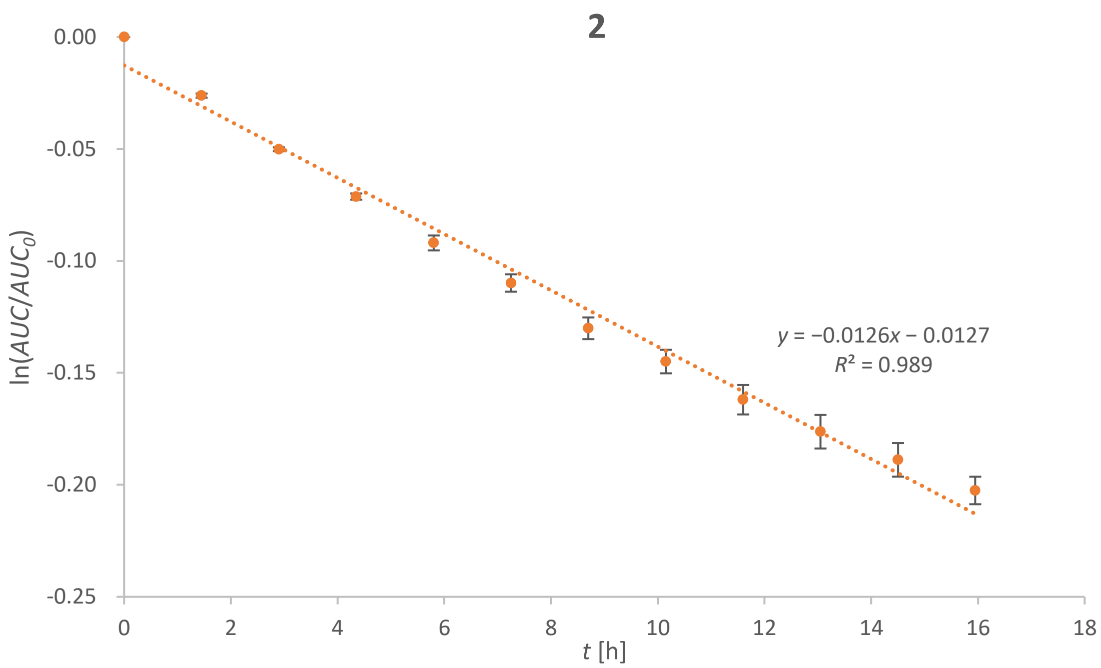

Figure S9. GSH stability assay of compound **2.** Error bars represent the standard deviation of triplicate measurements. AUC: area under the curve of residual starting material **2**, GSH: glutathione, t: time in hours.

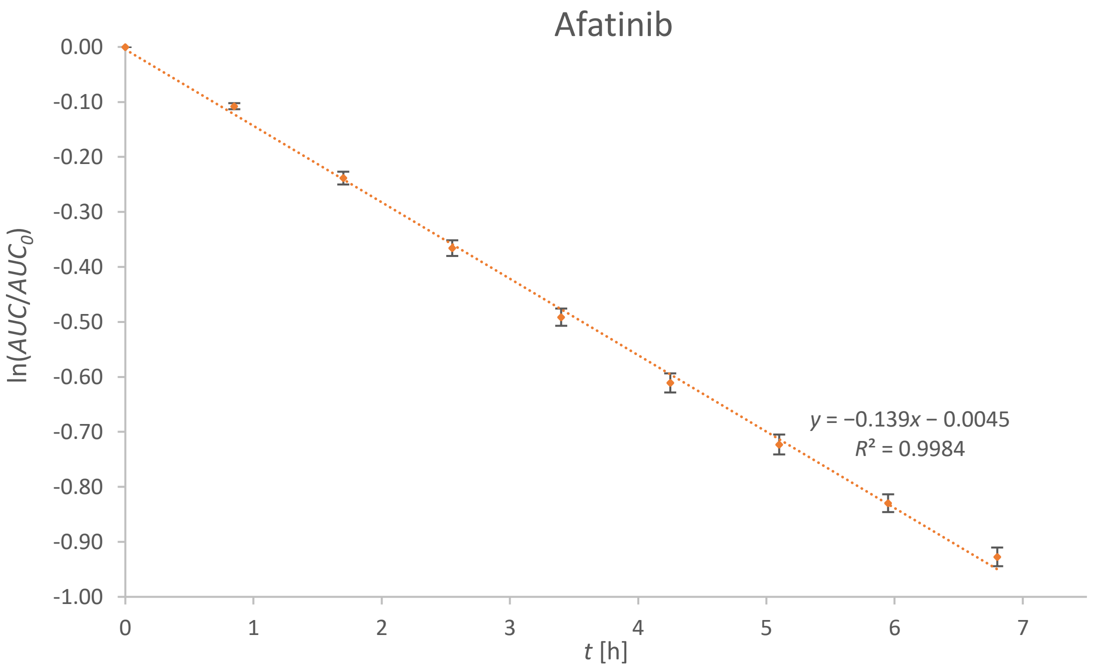

Figure S10. GSH stability assay of Afatinib. Error bars represent the standard deviation of triplicate measurements. AUC: area under the curve of residual starting material Afatinib, GSH: glutathione, t: time in hours.

4. *Nα*-Acetyl Lysine Stability Assay

HPLC specifications: Column: Phenomenex Kinetex 2.6 µM C8 100 Å 150 × 4.6 mm
Injection volume: 5 µL flow rate: 0.5 mL/min at 23 °C
HPLC-run **A** for **2**:

- 0 min: 40% MeOH, 60% phosphate buffer pH 2.3;
- 15 min: 85% MeOH, 15% phosphate buffer pH 2.3;
- 20 min: 85% MeOH, 15% phosphate buffer pH 2.3;
- 22 min: 40% MeOH, 60% phosphate buffer pH 2.3;
- 28 min: 40% MeOH, 60% phosphate buffer pH 2.3.

The half-lives $t_{1/2}$ for title compound **2** at pH 7.4 and pH 10.2 were calculated to be 1155 h and 27 h. The stability towards *Nα*-acetyl lysine at pH 10.2 was corrected for the slope of the curve of the aqueous degradation at pH 10.2 ($t_{1/2}$ = 139 h).

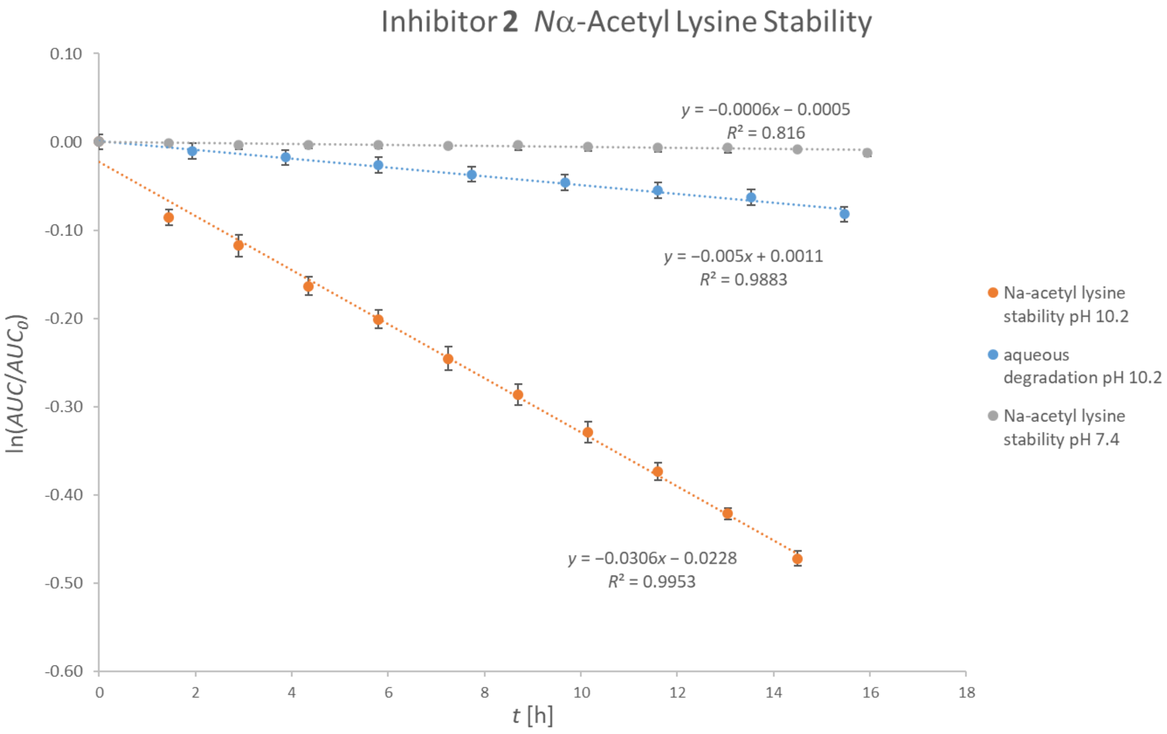

Figure S11. *Nα*-acetyl lysine stability assay of compound **2** at pH 7.4 and pH 10.2. Error bars represent the standard deviation of triplicate measurements. AUC: area under the curve of residual starting material **2**, t: time in hours.

5. Biochemical Assays

The IC_{50} curves for the kinases inhibited by title compound **2** (SGM373) are depicted below.

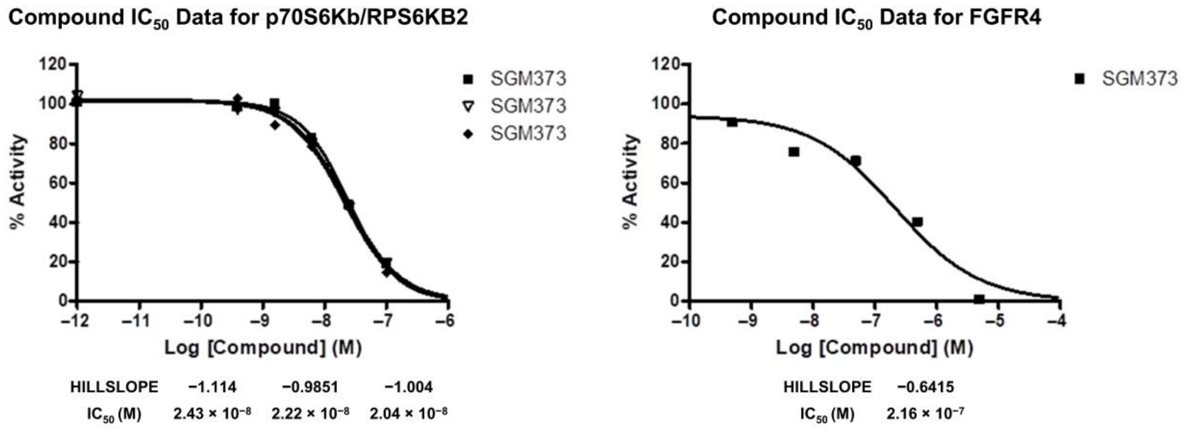

Figure S12. IC_{50} curves for the title compound **2.**

6. Microsomal Stability

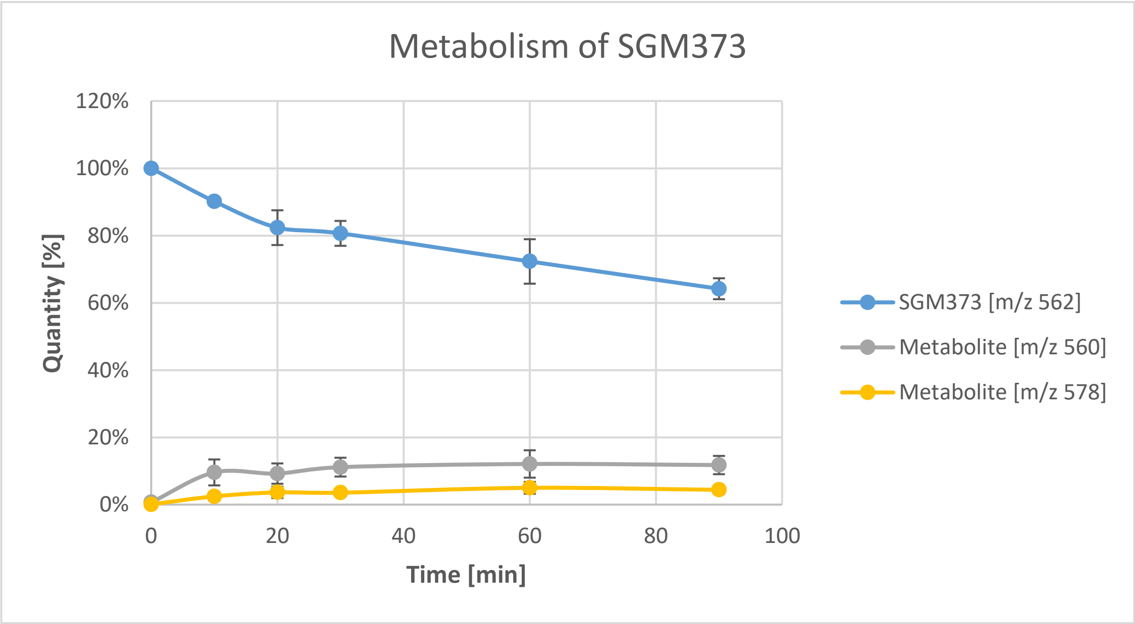

Figure S13. Microsomal stability of compound **2** (SGM373).

7. Thermal Shift Kinome Scan

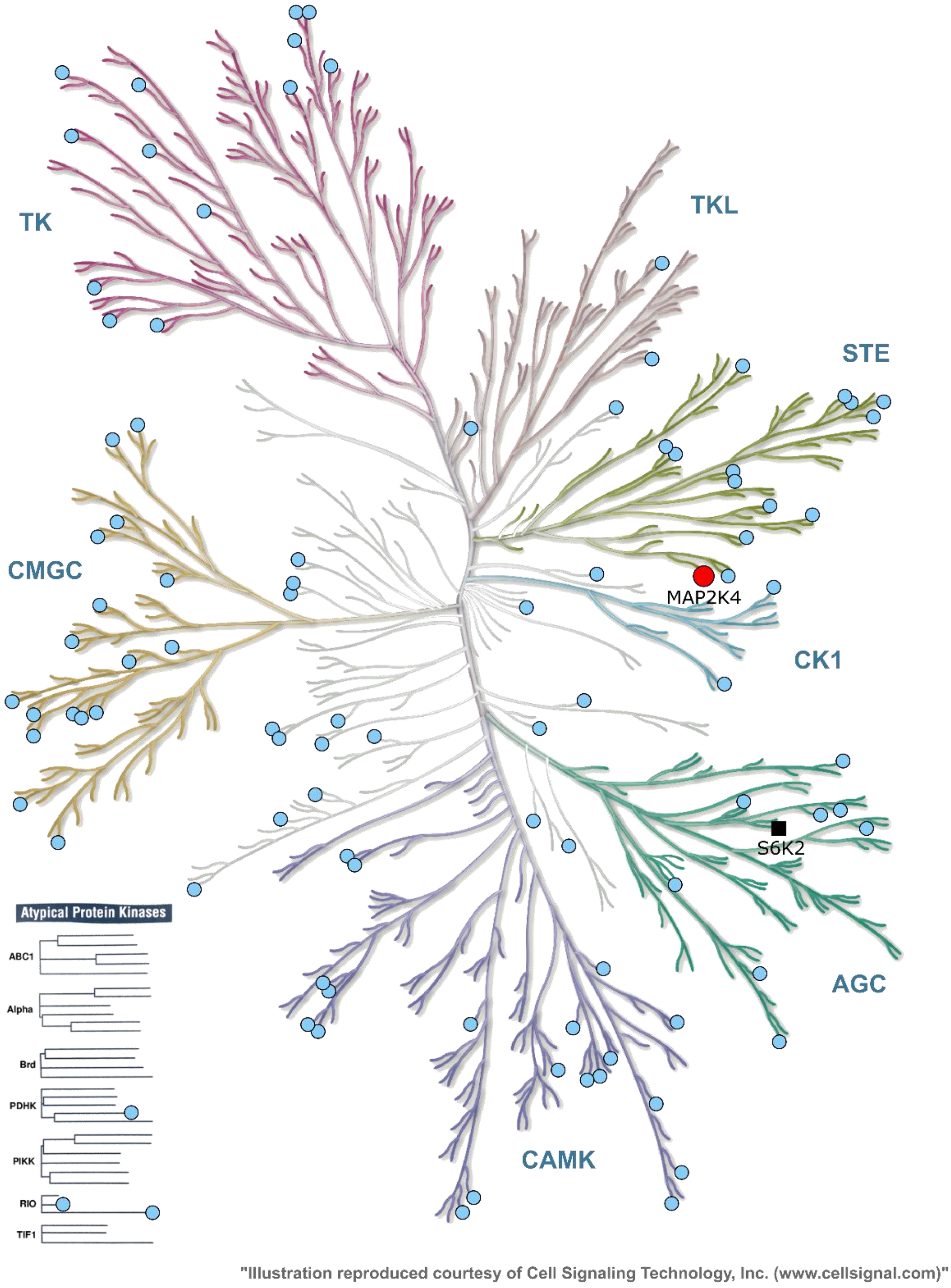

Figure S14. Protein kinases assessed in the Differential Scanning Fluorimetry (DSF) screening panel are distributed throughout the human kinome**.** Enzymes used for the assay are highlighted as blue dots in the dendrogram of the human kinome. Potential off-targets (ΔT_m > 2 °C) are marked as red dots. The target kinase is highlighted as a black square. The graphic was generated with KinMap (http://www.kinhub.org/kinmap/, accessed on 22 September 2021). AGC: containing PKA, PKG, PKC families, CAMK: calcium/calmodulin-dependent protein kinases, CK1: casein kinase 1, CMGC: containing CDK, MAPK, GSG3, CLK families, STE: homologs of yeast sterile 7, sterile 11, sterile 20 kinases, TK : tyrosine kinase, TKL : tyrosine kinase-like, MAP2K4: dual specificity mitogen-activated protein kinase kinase 4 (MKK4) , S6K2: ribosomal protein S6 kinase beta-2.

Table S1. Protein kinase thermal shift assay with ΔT_m for title compound **2**.

**Kinases**	**2 [ΔT_m]**	**Staurosporine [ΔT_m]**
MAP2K4	3	12.5
SLK	1.9	20.8
STK10	1.8	24.4
RPS6K5	1.5	15.0
TTK1	1.5	12.4
CAMK1D	1.1	10.5
MARK3	0.9	19.2
DAPK3	0.9	18.7
BRPF1	0.9	1.3
PAK4	0.8	14.9
STK39	0.6	9.3
ABL1	0.6	10.3
CAMKK2	0.6	25.1
RPS6KA6	0.6	1.0
EPHA7	0.5	12.7
EPHA5	0.5	8.7
MAP2K6	0.5	12.5
FGFR1	0.4	6.0
CHEK2	0.4	18.3
ULK3	0.4	17.4
OSR1	0.4	7.6
MELK	0.4	14.1
GSK3B	0.4	12.2
CSNK2A2	0.4	5.7
EPHA2	0.3	7.8
CDC42BPA	0.3	2.2
FGFR2	0.3	8.6
MAPK8	0.3	7.7
CLK1	0.3	13.7
RPS6KA1	0.3	4.6
MAPK6	0.2	1.0
PHKG2	0.2	22.5
AKT3	0.2	7.8
MAP3K5	0.2	17.6
DMPK1	0.2	9.8
DCAMKL1	0.2	6.3
STK6	0.2	17.3
RIOK1	0.2	−0.2
PLK4	0.2	19.0
NEK7	0.2	−0.2
CSNK1D	0.2	2.5
CLK3	0.1	7.5
DYRK2	0.1	7.0
CAMK4	0.1	8.9
FGFR3	0.1	13.7
MAPK10	0.1	8.2
TAF1	0.1	0.4
NEK2	0.1	2.2
MARK4	0.1	18.3
FLT1	0.1	12.5
CK2A1	0.1	4.4
GSG2	0.1	7.9
SRPK1	0	7.4

TLK1	0	8.9
BMP2K	0	19.2
MAPK9	0	4.8
AURKB	0	14.1
FES	0	7.5
MERTK	−0.1	5.9
CAMK2D	−0.1	17.5
BRAF	−0.1	0.4
PDK4	−0.1	0.1
MAPK15	−0.2	14.2
AAK1	−0.2	12.2
STK3	−0.2	14.8
STK38L	−0.2	11.3
PCTK1	−0.2	8.9
CDK2	−0.2	15.3
MAPK1	−0.2	−0.4
MAPKAPK2	−0.3	3.4
MST3	−0.3	6.5
MAP2K1	−0.3	1.6
CDKL1	−0.4	4.0
BMPR2	−0.4	2.9
VRK1	−0.4	3.4
SRC	−0.5	5.1
MAPK14	−0.5	0.0
DAPK1	−0.5	9.6
STK4	−0.5	15.5
TOPK	−0.5	8.1
PAK1	−0.5	7.4
BMX	−0.5	7.2
PIM1	−0.6	11.5
CAMK1G	−0.6	10.5
GAK	−0.6	9.5
MAPK13	−0.6	6.2
PIM3	−0.6	18.4
GPRK5	−0.6	8.6
STK17	−0.7	12.4
NEK1	−0.7	−0.7
STK17B	−0.8	8.6
RIOK2	−0.8	2.3
EPHB3	−0.9	6.7
WNK1	−0.9	1.4
CASK	−0.9	5.1
PKMYT1	−1	−1.1
BRD4	−1	−0.8
MST4	−1.1	4.6
CAMK2B	−2.3	11.4
DYRK1A	−2.4	10.7

References

1. Niwa, H.; Mikuni, J.; Sasaki, S.; Tomabechi, Y.; Honda, K.; Ikeda, M.; Ohsawa, N.; Wakiyama, M.; Handa, N.; Shirouzu, M.; et al. Crystal structures of the S6K1 kinase domain in complexes with inhibitors. *J. Struct. Funct. Genomics* **2014**, *15*, 153–164, doi:10.1007/s10969-014-9188-8.
2. Bienert, S.; Waterhouse, A.; de Beer, Tjaart A.P.; Tauriello, G.; Studer, G.; Bordoli, L.; Schwede, T. The SWISS-MODEL Repository—new features and functionality. *Nucleic Acids Res.* **2017**, *45*, D313–D319, doi:10.1093/nar/gkw1132.
3. Wang, J.; Zhong, C.; Wang, F.; Qu, F.; Ding, J. Crystal structures of S6K1 provide insights into the regulation mechanism of S6K1 by the hydrophobic motif. *Biochem. J.* **2013**, *454*, 39–47, doi:10.1042/BJ20121863.
4. Dermatakis, A.; Kabat, M.M.; Luk, K.-C.; Rosmann, P.L.; So, S.-S. Pyrimido[4,5-D]pyrimidine Derviatives With Anticancer Activity. Patent WO2004041822A1, 21 May 2004.

© 2021 by the authors. Licensee MDPI, Basel, Switzerland. This article is an open access article distributed under the terms and conditions of the Creative Commons Attribution (CC BY) license (http://creativecommons.org/licenses/by/4.0/).
